# HIV-1 Vpr induces ciTRAN to prevent transcriptional repression of the provirus

**DOI:** 10.1126/sciadv.adh9170

**Published:** 2023-09-06

**Authors:** Vipin Bhardwaj, Aman Singh, Aditi Choudhary, Rishikesh Dalavi, Lalchhanhima Ralte, Richard L. Chawngthu, Nachimuthu Senthil Kumar, Nagarjun Vijay, Ajit Chande

**Affiliations:** ^1^ Molecular Virology Laboratory, Department of Biological Sciences, Indian Institute of Science Education and Research (IISER), Bhopal, India.; ^2^ Synod Hospital Durtlang, Aizawl, Mizoram, India.; ^3^ Mizoram State AIDS Control Society (MSACS), Aizawl, Mizoram, India.; ^4^ Department of Biotechnology, Mizoram University, Aizawl, Mizoram, India.; ^5^ Computational and Evolutionary Genomics Laboratory, Department of Biological Sciences, Indian Institute of Science Education and Research (IISER), Bhopal, India.

## Abstract

The functional consequences of circular RNA (circRNA) expression on HIV-1 replication are largely unknown. Using a customized protocol involving direct RNA nanopore sequencing, here, we captured circRNAs from HIV-1–infected T cells and identified ciTRAN, a circRNA that modulates HIV-1 transcription. We found that HIV-1 infection induces ciTRAN expression in a Vpr-dependent manner and that ciTRAN interacts with SRSF1, a protein known to repress HIV-1 transcription. Our results suggest that HIV-1 hijacks ciTRAN to exclude serine/arginine-rich splicing factor 1 (SRSF1) from the viral transcriptional complex, thereby promoting efficient viral transcription. In addition, we demonstrate that an SRSF1-inspired mimic can inhibit viral transcription regardless of ciTRAN induction. The hijacking of a host circRNA thus represents a previously unknown facet of primate lentiviruses in overcoming transmission bottlenecks.

## Teaser

HIV-1 hijacks a host-encoded circular RNA to promote its transcription.

## INTRODUCTION

Circular RNA (circRNA) expression pattern is altered during immunological signaling, inflammation, and viral infection (*1*). However, very few circRNAs are known to exert regulatory influences in the context of immune function and viral infection (*2*–*7*). In particular, the role of circRNA in HIV-1 replication remains elusive.

CircRNAs are formed by back-splicing events that can occur in both coding and noncoding linear transcripts (*8*). The back-splice junction (BSJ) connects two ends and distinguishes a circRNA from its linear counterpart. By detecting and quantifying the number of distinct BSJ sequences, cDNA-based high-throughput sequencing approaches can be used to characterize circRNA expression in various pathological conditions (*9*, *10*) but are unable to detect circRNA BSJs in their native RNA form. While sequencing platforms, such as Oxford Nanopore Technologies (ONT), enable direct RNA sequencing (DRS), their potential currently remains limited to capturing polyadenylated transcripts (*11*). Accordingly, long-read sequencing technology has been applied to circRNAs only from reverse-transcribed products (*12*–*15*).

The detection of circRNAs using high-throughput sequencing thus often relies on reverse-transcribed products. However, the polymerase’s ability to switch templates can make subsequent validation difficult (*16*, *17*). In addition, polymerase chain reaction (PCR) bias can lead to an overrepresentation of certain circRNAs, and other factors such as trans-splicing and exon repetitions can add complexity to the analysis of circRNA expression (*18*). In the context of viral infections, the abundance of viral transcripts, generation of fusion transcripts, and the overall deregulation of RNA metabolism can obscure host-encoded, less-abundant RNAs, making it necessary to explore alternative methods for detecting low-abundant RNA species such as circRNAs during infection (*19*). In this study, we present a pipeline for detecting circRNAs using nanopore direct RNA sequencing (DRS) (circDR-seq) and report a cellular function for circSMARCA5, which is here denoted as ciTRAN owing to its ability to modulate HIV-1 transcription.

## RESULTS

### Enrichment of circRNAs for direct RNA nanopore sequencing

**Fig. 1.**
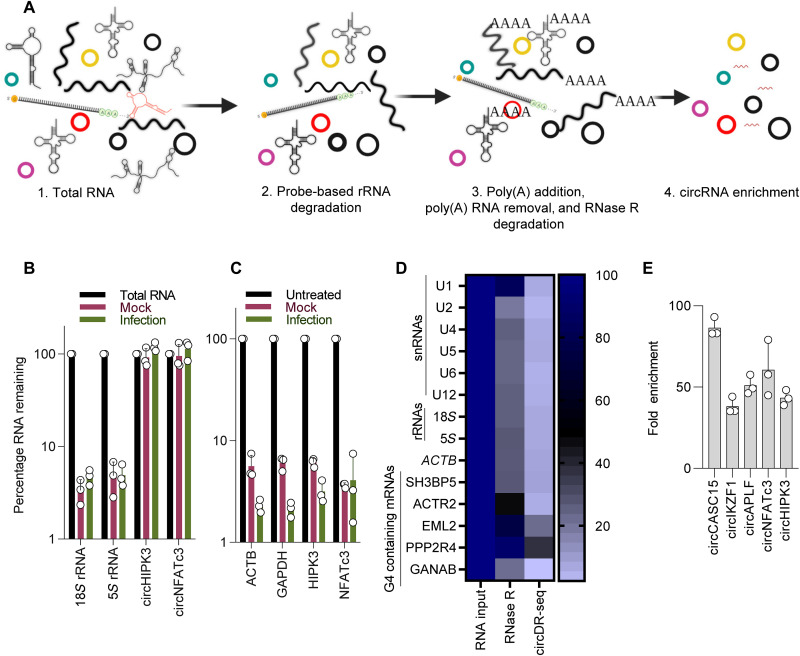
circRNA enrichment by iterative depletion of linear RNA pool. (**A**) Schematics depicting sequential steps to deplete the total RNA of linear RNAs and enrich circRNA fraction. (**B**) Reverse transcription quantitative PCR (RT-qPCR) for residual 18*S* and 5*S* rRNAs and the indicated circRNAs post-rRNA depletion. The data are normalized to *GAPDH*. (**C**) Residual mRNAs after poly(A) depletion and data normalized to circHIPK3 and cirNFATc3. (**D**) Comparison of RNase R alone and circDR-seq for assessing levels of subsets of snRNAs, G4 region containing mRNAs, and rRNAs after respective treatments of the mock-treated RNA sample. Data obtained by RT-qPCR are normalized to circHIPK3. (**E**) Extent of circRNA enrichment assessed by RT-qPCR of mock-treated sample for the indicated candidate circRNAs. Data are normalized to glyceraldehyde-3-phosphate dehydrogenase (GAPDH). *n* = 3; ±SD.

The successful detection of circRNA through DRS relies heavily on the effectiveness of circRNA isolation approach in a context where linear RNA exceeds in abundance by more than 99%. Therefore, we sought to sequentially remove non-circRNAs by iterative depletion of the linear RNA species to enrich circRNA pool (Fig. 1A). Accordingly, total RNA from the mock and HIV-1–zsGreen (details in the Supplementary Materials) infected Jurkat E6.1T cells (fig. S1A) was first subjected to ribosomal RNA (rRNA) depletion by probe-based degradation by ribonuclease (RNase) H (fig. S1B), which led to more than 95% reduction of rRNA species (18*S* and 5*S* rRNA) without affecting the representative circRNAs (Fig. 1B). The remainder linear pool from the ribo-depleted fractions was first polyadenylated using *Escherichia coli* poly(A) polymerase and subsequently removed using oligo(dT)–coated magnetic beads (fig. S1C), leading to the depletion of more than 94 and 97% of the abundant linear RNAs from the mock and infected RNA pool, respectively (Fig. 1C). The efficiency of RNase R is limited by the presence of structural RNAs and the availability of diverse substrates, including small nuclear RNAs (snRNAs) (*20*). We found that, when compared to the RNase R treatment alone, circDR-seq enabled the efficient depletion of G-quadruplexes containing RNAs (G4-RNA) and the small RNA fraction (Fig. 1D) while resulting in a 50- to 86-fold enrichment of representative circRNAs species (Fig. 1E).

### Detection of circRNAs in the native form and associated modifications

**Fig. 2.**
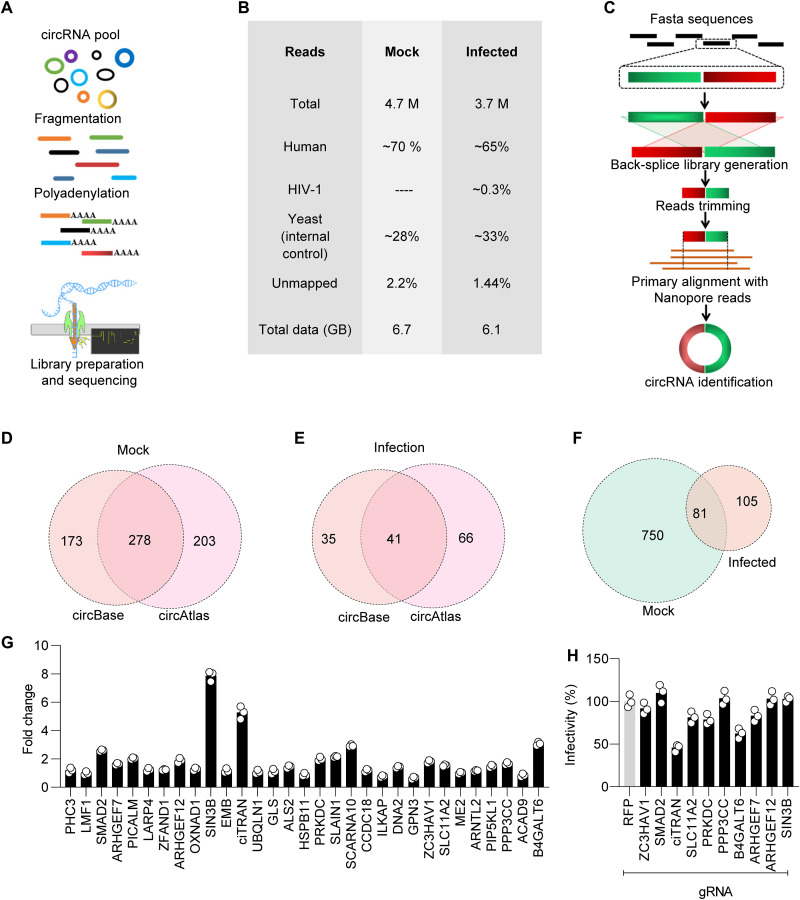
Nanopore DRS reveals infection-induced circRNAs. (**A**) Schematics depicting processing of enriched circRNA pools for nanopore DRS (method detailed in the Supplementary Materials). (**B**) Table showing mapped reads obtained from three flow cells per sample after high-precision basecalling. (**C**) Schematics showing a computational pipeline developed for the detection of BSJs using circBase, circAtlas, and virtual exonic circRNA library. (**D** and **E**) Number of circRNAs obtained using the circBase-specific library in mock (D) and infection (E). (**F**) Numbers of circRNAs obtained after analysis of virtual exonic circRNA library using a pipeline in (C). (**G**) Infection-specific high-confidence circRNA candidates validated using RT-qPCR from HIV-1–infected Jurkat E6.1 cells. Data were normalized to GAPDH. (**H**) Luciferase activity measured as a function of HIV-1 Luc infection from JTAg cells depleted of indicated circRNAs. *gRFP* served as nonrelevant guide RNA (gRNA). *n* = 3; ±SD. M, million; GB, gigabyte.

Because DRS by nanopore requires a linear polyadenylated RNA as an input, we linearized the enriched circRNAs through controlled fragmentation and the addition of a poly(A) tail (detailed in Materials and Methods), allowing for DRS (Fig. 2A). The addition of poly(A) tail to the fragmented circRNA species from Fig. 1E was confirmed by amplification from cDNA templates generated by oligo(dT) priming (fig. S1D). After confirming the addition of poly(A) tails, we generated the library from three replicates and obtained the raw reads. Of the raw reads obtained (Fig. 2B), 65 to 70% were found to align with the human transcriptome, with the remaining 28 to 33% being attributed to an internal standard of yeast enolase RNA included in the RNA sequencing kit. Next, we used NanoPack, a tool for visualizing and analyzing nanopore sequencing data, to examine the raw data obtained from both mock and infected samples. Our analysis showed that the read length, total yield, base calling accuracy, and average read quality were comparable between the two sets of samples (fig. S2, A to H; analysis detailed in the Supplementary Materials).

Next, to identify BSJs in the sequencing data, we extracted FASTA sequences of known circRNAs from the circBase and circAtlas databases and used a pipeline outlined in Fig. 2C to join the ends of these sequences and obtain BSJ sequences. Considering the precision, recall, and F1 score, as shown in fig. S3A, we chose to use a 100–base pair (bp) library for pblat (as outlined in Fig. 2C and the Supplementary Materials). By applying these criteria, we were able to identify 451 and 481 circRNAs, with a shared total of 278 circRNAs present in the sample treated with mock conditions (Fig. 2D). In infected cells with an overlap of 41 circRNA, 76 and 107 circRNAs were detected (Fig. 2E). The number of circRNAs was comparatively less in HIV-1 infection, which might have resulted from the induction of cellular RNAses (*2*). We went beyond using pre-existing databases to detect circRNAs by creating a virtual exonic library. This approach revealed a number of previously unannotated circRNAs, with 151 found in the mock-treated sample and 39 in the infected sample (fig. S3B). We also verified the presence of previously unannotated BSJs by using Sanger sequencing to confirm two unannotated circRNAs (fig. S3, C and D). Consistent with the higher prevalence of the circRNAs from the coding region (*21*–*23*), we found more than 80% of the circRNAs originating from protein-coding genes (fig. S3, E and F). Furthermore, a comparison of the size distribution and strandedness (positive or negative DNA strand) corroborated with circBase (*24*), eliminating any size and strand-based bias in detection by circDR-seq (fig. S3, G to K).

One of the advantages of DRS by nanopore is that it enables simultaneous detection of m6A modification. RNA m6A modification regulates key functions such as controlling RNA splicing, translation, stability, and innate immune response to infection (*25*). In the context of circRNAs, m6A modification can endow a coding potential (*26*). Considering that circDR-seq can allow the simultaneous detection of m6A RNA modification, we assessed this possibility, in addition to internal ribosome entry site (IRES) detection, to predict protein-coding circRNAs. Through an integrative analysis (using tools like Nanom6A, CPC2, and IRES finder), we obtained high-confidence protein-coding circRNA candidates. Nanom6A revealed 120 and 21 m6A containing sites that comprised 28 and 7 circRNAs, respectively (fig. S4, A and B). In addition, randomly selected candidates were also validated for the presence of m6A (fig. S4, C and D). Further, CPC2 and IRES analysis showed an overlap of 98 and 16 circRNAs in mock and infection, respectively (fig. S4, E and F). Four overlapping circRNAs harboring m6A were detected by CPC2, likely indicating circRNAs with coding potential (fig. S4G). Despite not finding a distinct profile in these experimental conditions, the validation experiments showed the capability of circDR-seq in detecting circRNA modifications simultaneously.

Last, to check the robustness in circRNA detection, we compared circDR-seq with CIRI-long (*13*) and circNICK-LRS (*15*). At par with these recently developed tools, the comparison showed 99.93 and 82.20% efficiency, respectively (fig. S4, H to K). Despite generating fewer reads than Illumina sequencing run (which can generate >60 to 100 million reads), the number of circRNAs detected by circDR-seq appears to be considerable (from ~4 million reads). By increasing the number of flow cells, we believe it is possible to generate equivalent reads to an Illumina run for a more comprehensive comparison of these orthogonal platforms. Overall, using circDR-seq, 1017 circRNAs candidates (831 in mock and 186 in infection) were detected in their native form (Fig. 2F), and the possibility of simultaneously capturing RNA modifications and putative coding potential was also assessed.

### Infectivity inhibition concomitant with ciTRAN depletion in T cells

To understand the functions of circRNAs in HIV-1–infected T cells, we narrowed down a list of 41 candidates based on their presence in two databases (Fig. 2E). Of these, five were also present in uninfected cells and were excluded from further analysis. Of the remaining 36 candidates, referred to as infection specific, we obtained a reliable PCR product of the desired size for 31 circRNAs. Of these 31, the expression levels of 10 circRNAs were at least 1.5 times higher in infected cells than in uninfected cells (Fig. 2G). We hypothesized that these up-regulated circRNAs may play a role in the course of HIV-1 infection. To investigate the potential functions of the circRNAs detected in HIV-1–infected cells, we designed guide RNAs (gRNAs) targeting the BSJs of select candidates (from Fig. 2G) and performed CRISPR-CasRx-based knockdown in Jurkat T cell [Jurkat TAg (JTAg)] cells (fig. S5A). The cells depleted of the indicated circRNAs were selected on blasticidin and expanded, and the knockdown for each circRNA was confirmed using reverse transcription quantitative PCR (RT-qPCR) (fig. S5B; circRNA-specific primers listed in auxiliary data table S1). When these circRNA-depleted cells were infected with HIV-1 (HIV-1-Luc; details in the Supplementary Materials), we observed that ciTRAN (55%) and, to a lesser extent, circB4GALT6 (37.5%) reduced the expression of luciferase encoded by the virus (Fig. 2H). The gRNA-targeting ciTRAN (circSMARCA5) was specific to the circRNA, and the cognate linear RNA (*SMARCA5*), as well as protein (SMARCA5) levels, remained unaffected in these conditions (fig. S5, C to E). Because of its greater impact on viral infectivity, we focused on further examining the role of ciTRAN in HIV-1 infection.

### ciTRAN relieves a post-integration block to viral gene expression

**Fig. 3.**
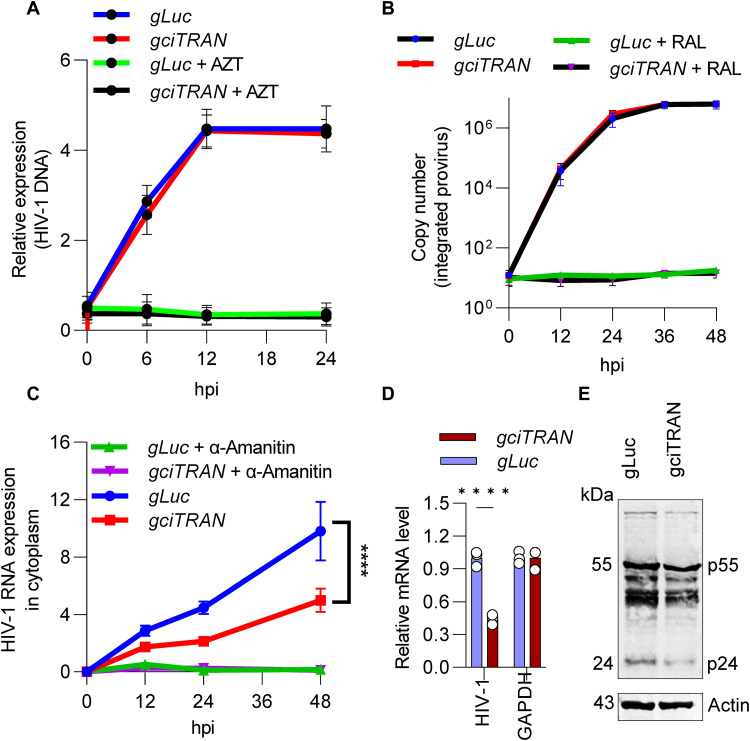
A post-integration event is targeted by ciTRAN. Effects of ciTRAN knockdown during HIV-1–zsGreen infection on: (**A**) reverse transcription products as assessed by measuring cytoplasmic HIV-1 DNA (AZT served as a control), (**B**) proviral integration as assessed by Alu-Gag PCR for HIV-1 genomic DNA integration [raltegravir (RAL) served as a control], and (**C**) cytoplasmic HIV-1 RNA (*gag*) expressed from the provirus (α-amanitin served as a control). Hours post-infection (hpi). (**D**) Nuclear transcription (*gag*) from an integrated provirus in ciTRAN depleted condition assessed by nuclear run-on assay. GAPDH served as control. gRNA to luciferase served as a nonrelevant gRNA. (**E**) Immunoblot indicating intracellular viral Gag levels; actin served as a loading control. *n* = 3; ±SD.

To determine the stage of the virus life cycle targeted by ciTRAN, we used HIV-1–zsGreen–infected cells that either expressed a nonrelevant gRNA (Luciferase gRNA) or a ciTRAN-specific gRNA. We collected subcellular fractions from these infected cells and used them to measure reverse transcription products (HIV cDNA), integration events (proviral integrants), and transcription from the provirus (HIV cytoplasmic mRNA) (*27*). We found that when ciTRAN was depleted, the amount of reverse-transcribed HIV cDNA did not change (Fig. 3A), nor did the number of integrated provirus (Fig. 3B). However, the amount of cytoplasmic viral mRNA was reduced (Fig. 3C), and a nuclear run-on assay further revealed a 2.3-fold reduction (Fig. 3D). These decreases in transcripts resulted in a reduction of intracellular viral Gag accumulation, as shown by Western blotting from the infected cell lysates (Fig. 3E), indicating that ciTRAN affects viral gene expression.

### ciTRAN binds to a negative regulator of HIV transcription

**Fig. 4.**
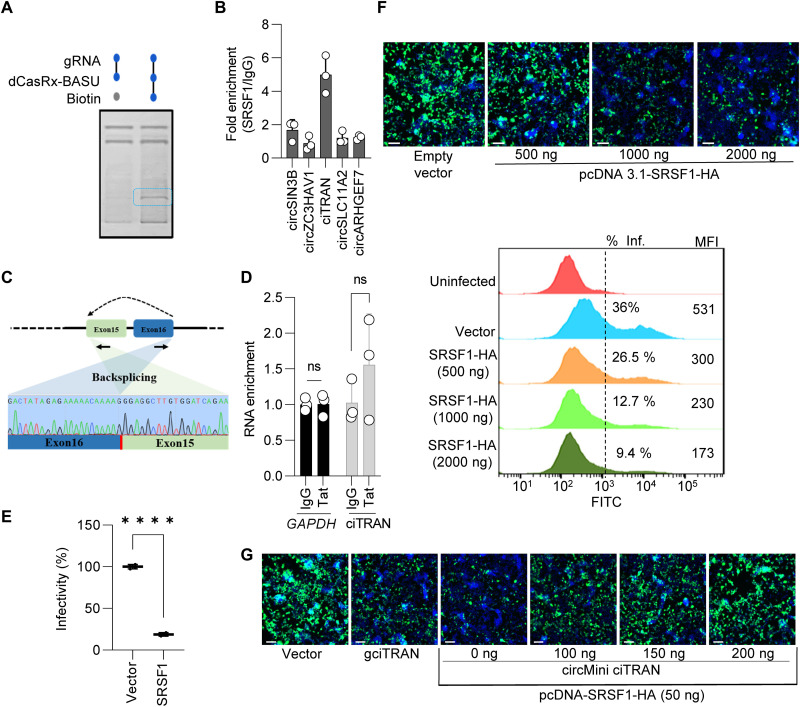
ciTRAN interacts with a negative regulator of HIV-1 infectivity. (**A**) Biotinylated protein fractions obtained by ciTRAN-mediated dCasRx-BASU–based proximity labeling visualized by silver staining. The differentially resolved band is highlighted. (B) SRSF1-mediated PAR-CLIP followed by qPCR for indicated circRNA targets. (GAPDH was used for normalization); *n* = 3; ±SD. (**C**) Back-splicing junction of enriched ciTRAN RNA from (B) was validated using Sanger sequencing. (**D**) HIV-1 Tat-mediated PAR-CLIP followed by qPCR using ciTRAN and GAPDH-specific primers; *n* = 3; ±SD. (**E**) Effect of SRSF1 ectopic expression on HIV-1 infectivity in JTAg target cells; *n* = 3; ±SD. JTAg cells were electroporated with pCDNA 3.1(−) and pCDNA 3.1(−)-SRSF1-HA and then were infected HIV-Luc. (**F**) HIV-1 produced by electroporation from JTAg cells along with either empty vector or increasing concentrations of SRSF1 (pCDNA 3.1 SRSF1-HA: 500, 1000, and 2000 ng) and subsequent infectivity in TZM-GFP target cells captured using microscopy [top: scale bars, 100 μm (representative images)], and flow cytometry (bottom: Inf, infectivity expressed as percentage of zsGreen-positive cells; MFI, mean fluorescence intensity). (**G**) Effect of ciTRAN knockdown, ciTRAN ectopic expression [pCDNA 3.1(+) circMini ciTRAN: 100,150, and 200 ng], and SRSF1 expression [pCDNA 3.1(−)–SRSF1–HA: 50 ng] on HIV-1 infectivity. HIV-1 produced from HEK293T in the presence of either vector, gciTRAN (ciTRAN knockdown cells), synthetic ciTRAN-expressing vector [pCDNA 3.1(+) circMini ciTRAN], or SRSF1 expression vector [pCDNA 3.1(−)–SRSF1–HA]. Representative images of infected TZM-GFP target cells (scale bars, 100 μm) under indicated conditions. ns, not significant; IgG, immunoglobulin G; FITC, fluorescein isothiocyanate.

CircRNAs can regulate parental gene expression owing to their ability to base-pair with DNA or RNA and can establish specific interactions with proteins (*8*). Furthermore, circRNAs can also directly establish interactions with chromatin or regulate parental gene expression by binding to protein mediators of transcription (*28*, *29*). Our experiments thus far suggested the association of ciTRAN depletion with reduced transcription from the provirus. To understand the mechanism underlying this regulation, we used the CARPID (CRISPR-assisted RNA-protein interaction detection method) (*30*). Accordingly, specific guides were generated that installed a catalytically dead CasRx fused with biotin ligase on the BSJ of ciTRAN. A pulse labeling of biotin followed this, subsequently enabling the enrichment of a biotinylated pool using a streptavidin affinity matrix (fig. S6A). The biotinylated proteins were then resolved on SDS–polyacrylamide gel electrophoresis (SDS-PAGE), and differentially enriched bands were isolated and subjected to mass spectrometry. Mass spectrometry analysis revealed SRSF1 as a top candidate interactor of ciTRAN (Fig. 4A and fig. S6B). Further verification by PAR-CLIP (photoactivatable ribonucleoside–enhanced crosslinking and immunoprecipitation) from the lysates of infected T cells, reciprocally, showed a sixfold enrichment of ciTRAN but not other indicated circRNAs, with SRSF1-specific antibody over immunoglobulin G (IgG) control (Fig. 4B). We also verified the enriched BSJ by Sanger sequencing, further confirming the specificity of this interaction (Fig. 4C). HIV-1 Tat being a major stimulator of HIV-1 transcription, Tat protein binding to circRNA could have resulted in repression of the viral transcription. Therefore, we performed Tat PAR-CLIP to examine this. Our experiments ruled out the possibility of ciTRAN sequestering Tat (Fig. 4D) and concurrently modulating viral transcription. These experiments suggest that ciTRAN possibly regulate HIV-1 transcription via its interaction with the host protein SRSF1.

JTAg T target cells ectopically expressing hemagglutinin (HA)–tagged SRSF1 phenocopied the effects of ciTRAN down-regulation (Fig. 4E). In addition, SRSF1 expression in the producer JTAg cells also correspondingly reduced the number of infectious progeny virions, as revealed by infectivity assay in target TZM–green fluorescent protein (GFP) cells (Fig. 4F and fig. S6C). These experiments suggest an impact during the viral biogenesis and subsequent progeny virion infectivity (Fig. 4, E and F). Conversely, we demonstrated that depletion of SRSF1 in JTAg cells leads to an augmentation in long terminal repeat (LTR) transactivation activity, as illustrated in fig. S6D. Furthermore, HIV-1–zsGreen infectivity in human embryonic kidney (HEK) 293T target cells ectopically expressing SRSF1 mirrored that of cells lacking ciTRAN (Fig. 4G and fig. S6E), and, notably, this inhibition by SRSF1 protein was reversed by synthetic ciTRAN expressor (pCDNA 3.1(+) circMini ciTRAN) (Fig. 4G). To rule out gRNA-specific effects, we also reproduced these findings by using two different short hairpin RNAs (shRNAs) targeting ciTRAN, confirming the specificity of the results associated with ciTRAN knockdown by CRISPR-CasRx (fig. S6, F and G).

SRSF1 being a splicing factor, we generated SRSF1 knockdown cells to explore the interdependency between SRSF1 and ciTRAN levels, and intriguingly, we observed no substantial change in the level of ciTRAN upon SRSF1 knockdown (fig. S6, H and I). However, previous studies have indicated the involvement of quaking (QKI) in ciTRAN biogenesis (*31*). Therefore, we generated QKI knockdown cells and found that QKI, rather than SRSF1, is responsible for regulating ciTRAN biogenesis (fig. S6, J and K). Because SRSF1 is involved in RNA splicing, we conjectured that conditions that affect the local availability of SRSF1, such as ciTRAN-mediated sequestration, would affect RNA splicing and concurrently regulate HIV-1 replication. Therefore, we looked into select SRSF1-regulated splicing events that have been implicated in HIV replication. The RNA sequencing data from SRSF1 wild-type and knockdown cells from a previous study (*32*) were analyzed to capture a subset of differentially spliced candidates relevant for HIV-1 infection. The splicing patterns of these genes were then examined from vector- and ciTRAN-overexpressed cells (a condition that will recapitulate SRSF1 protein availability and its impact on the splicing of host genes relevant for HIV-1 replication). While we observed some changes in splicing patterns that could have resulted from SRSF1 sponging by ciTRAN (fig. S7A), we speculate that these changes are unlikely to have a functional impact on HIV-1 biogenesis and subsequent rounds of infectivity because viral RNA transcription event precedes the former. Moreover, ectopic expression (20- to 50-fold) of SRSF1 may affect viral mRNA splicing because SRSF1 has been shown to play multiple roles in the biogenesis of HIV-1 mRNA and can modulate viral RNA splicing events. We thus speculated that SRSF1 activity regulation by ciTRAN overexpression might negatively affect viral RNA splicing. Consequently, we conducted experiments to assess the impact of ectopically expressing ciTRAN on HIV-1–spliced RNAs, including those encoding Gag, Vpu, and Vpr, as well as alternative splicing from multispliced 2-kb RNA class. Unexpectedly, we observed increased individual mRNA levels without any alterations in the splicing pattern (fig. S7, B and C).

In addition, we investigated the effect of ciTRAN knockdown on HIV-1–unspliced, single-spliced, and multispliced RNAs, which resulted in a decrease in the levels of HIV-1 RNA (fig. S7, D to F). These results and the experiments using LTR reporters (fig. S6D) counter the possibility that the inhibition of viral infectivity resulted from changes in the host or the viral RNA splicing.

### ciTRAN obstructs LTR accessibility of SRSF1 to promote infectivity

**Fig. 5.**
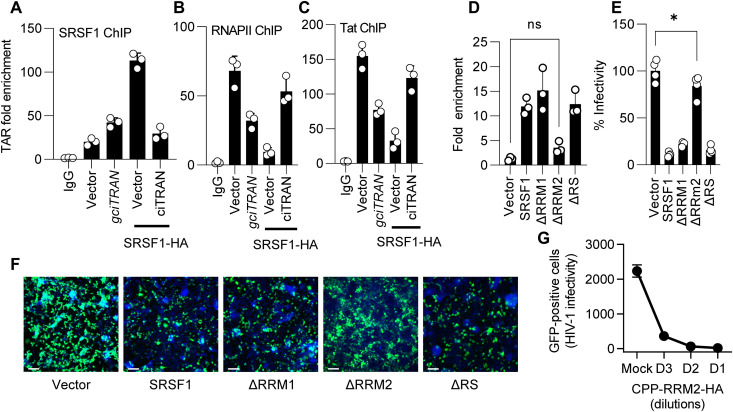
SRSF1 sequestration by ciTRAN promotes HIV-1 transcription. (**A** to **C**) ChIP analysis from JTAg cells and quantification of TAR locus enrichment by RT-qPCR for SRSF1 (A), RNAPII (B), and HIV-1 Tat (C); *n* = 3; ±SD. NL4-3 Env(−) Nef(−) vector was electroporated in the JTAg cells along with vector, gciTRAN, SRSF1 with empty vector pcDNA 3.1(+) circMini, and pCDNA3.1(+) circMini encoding ciTRAN. Line highlighting SRSF1 shows the SRSF1 overexpression state along with circMini empty vector and circMini encoding ciTRAN. The fold enrichment method was used to represent the data. (**D**) Effects of SRSF1 deletion mutants on ciTRAN interaction assessed by PAR-CLIP. SRSF1 deletion mutants were ectopically expressed through pCDNA 3.1 (−) HA construct in JTAg cells by electroporation, and PAR-CLIP was performed using an anti-HA antibody. (**E** and **F**) Effects of indicated SRSF1 deletion mutants on single-cycle HIV-1 infectivity (*n* = 4; ±SD) and representative images (F) from TZM-GFP cells (scale bars, 100 μm). HIV was produced from JTAg cells by electroporation, and the infection was given in TZM-GFP target cells and scored by counting green cells. (**G**) Effects of purified, serially diluted [D1(1:1), D2(1:10) and D3(1:100) (details in methods)] CPP-tagged SRSF1 mimic (CPP-RRM2) on HIV-1 infection assessed by scoring GFP-positive cells.

To gain a deeper understanding of how the ciTRAN-SRSF1 axis affects the viral gene expression, we performed chromatin immunoprecipitation (ChIP) experiments to examine the occupancy of SRSF1, RNAPII, and Tat on the HIV-1 promoter [trans-activation response element (TAR) locus]. ChIP experiments indicated that SRSF1 interaction with the TAR locus (Fig. 5A and fig. S8A) was enhanced by ciTRAN depletion. Strikingly, the enhanced SRSF1 protein association with the TAR locus (achieved by ectopic expression of SRSF1-HA) was reversed by overexpression of ciTRAN from a synthetic construct, suggesting a competing scenario (Fig. 5A). We also checked the occupancy of RNAPII on the HIV-1 promoter under these identical conditions. We found that increased SRSF1 association correspondingly excluded Pol-II from the viral promoter and that ciTRAN overexpression restored the polymerase association (Fig. 5B and fig. S8A). SRSF1 is known to replace HIV-1 Tat from the transcription complex (*33*). Our experiments resolve this by revealing a reduction in Tat’s interaction with the TAR locus in ciTRAN-depleted conditions (Fig. 5C and fig. S8A). These findings confirm that ciTRAN promotes active transcription from the viral promoter by obstructing SRSF1.

### RRM2-inspired mimic can intercept ciTRAN action

SRSF1 contains two RNA recognition motifs (RRMs) and a C-terminal rich in arginine/serine-rich (RS) (*33*, *34*). We next sought to determine the specific regions of SRSF1 that could interact with ciTRAN and how this interaction affects viral transcription. We used a series of deletion clones (fig. S8, B and C) and the PAR-CLIP technique to study this. Our results showed that when the RRM2 region of SRSF1 was deleted, there was no longer any enrichment of ciTRAN (Fig. 5D). This loss of interaction between ciTRAN and the ΔRRM2 mutant also concorded to an increase in viral infectivity (Fig. 5, E and F).

To further understand the potential of targeting the ciTRAN-SRSF1 axis, we examined the role of specific SRSF1 protein domains. Toward this, we developed a secretory, cell-penetrating peptide (CPP)–fused RRM2-HA. This cell-free CPP-tagged RRM2 obtained from the supernatant of transfected cells demonstrated the ability to bind to the TAR locus and ciTRAN as well as consequently inhibit viral replication, whereas RRM1-HA, expectedly, failed to inhibit LTR transactivation (Fig. 5G and fig. S8, D to G). These results highlight the potential of targeting the ciTRAN-SRSF1 axis to curtail the infection. Collectively, our findings also provide insight into the molecular features required by SRSF1 to recognize ciTRAN and the effects on viral replication.

### HIV-1 Vpr induces ciTRAN expression

**Fig. 6.**
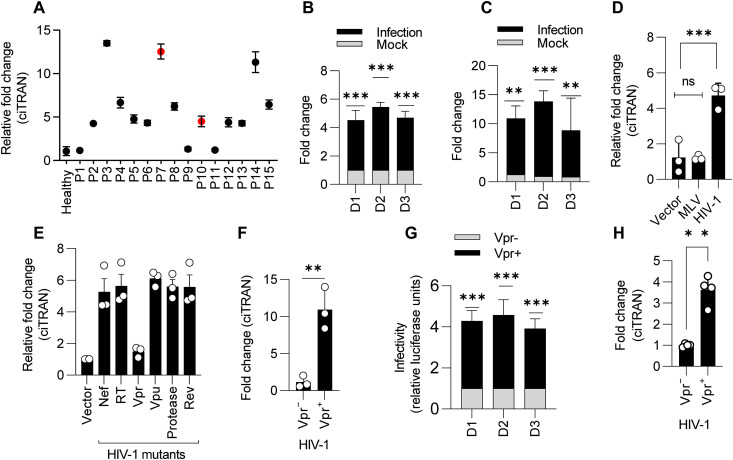
ciTRAN is induced by Vpr. (**A**) ciTRAN level analyzed using RT-qPCR from total RNA isolated from the whole blood of healthy donors (H; *n* = 5) and HIV-infected individuals (P; *n* = 15). Highlighted in red are not undergoing any ART. (**B** and **C**) Effect of HIV-1 infection on ciTRAN levels in the PBMCs (B) and CD4^+^ cells (C) isolated from three healthy donors (D1 to D3). HIV-1–zsGreen was used for infecting the isolated PBMCs and CD4^+^ cells for 48 hours. Subsequently, RNA from infected and mock-treated cells was used for ciTRAN level estimation by qRT-PCR; *n* = 3; ± SD. (**D**) Effects of MLV and HIV-1 on ciTRAN induction in JTAg cells; *n* = 3 ± SD. MLV encoding plasmid NCA zsGreen reporter and NLBN zsGreen reporter (HIV) were electroporated in JTAg cells, and RNA from these cells was used for qRT-PCR analysis after 48 hours after transfection for ciTRAN analysis. Data were normalized to GAPDH. (**E**) Effect of viral proteins on ciTRAN induction estimated using RT-qPCR after electroporation of JTAg cells with HIV-1–encoding plasmids lacking the indicated viral genes (plasmids described in Supplementary Table); *n* = 3 ± SD. (**F**) ciTRAN levels in CD4^+^ cells assessed by RT-qPCR after HIV-1 Luc Vpr(+/−) virus infection; *n* = 3; ±SD. HIV-1 produced with and without Vpr from HEK293T was used to infect CD4^+^ cells. (**G**) HIV-1 Luc Vpr(+/−) infectivity in CD4^+^ primary T cells. (**H**) ciTRAN level revealed by RT-qPCR in primary CD4^+^ cells infected with HIV-1 Vpr(+/−).

Building on our findings of the ciTRAN-SRSF1 axis in HIV-1 transcriptional regulation, we then sought to investigate the viral side of the equation. Given that SRSF1 is a constitutively expressed protein, we hypothesized that the virus has a mechanism to induce the circRNA to counteract its effects. Our experiments suggested an average 5.6-fold ciTRAN induction from natural infections (*n* = 15; Fig. 6A). In addition, when peripheral blood mononuclear cells (PBMCs) and purified primary CD4^+^ cells isolated from healthy donors were infected with HIV-1 (fig. S9, A to C), we observed up to 10-fold induction of ciTRAN (Fig. 6, B and C). Notably, ciTRAN up-regulation was limited to HIV-1, and murine leukemia virus (MLV) could not induce it, as revealed by the transfection of HIV-1– and MLV-encoding constructs in JTAg cells (Fig. 6D). These results implied the relevance of ciTRAN expression in HIV-1–infected primary cells and cell lines and further prompted us to surmise the involvement of virally encoded proteins in stimulating the ciTRAN expression. To find out, we examined ciTRAN levels by transfecting the virus genomes (as in Fig. 6D) that could not produce indicated viral accessory/regulatory proteins and enzymes. ciTRAN levels were unchanged in almost all conditions except when the virus lacked Vpr (Fig. 6E). The infection of primary CD4^+^ cells with isogenic viruses HIV-1 Luc (Vpr +/−) further confirmed the involvement of virion-encoded Vpr in ciTRAN induction (Fig. 6, F to H). Furthermore, we conducted additional investigations to assess the levels of SMARCA5 and SRSF1 at both the RNA and protein levels during Vpr ectopic expression to rule out any indirect effects. Our results revealed an induction of SMARCA5 at the mRNA level, while the protein levels remained unaffected (fig. S9D). For SRSF1, similar mRNA and protein levels upon Vpr expression were observed (fig. S9E).

**Fig. 7.**
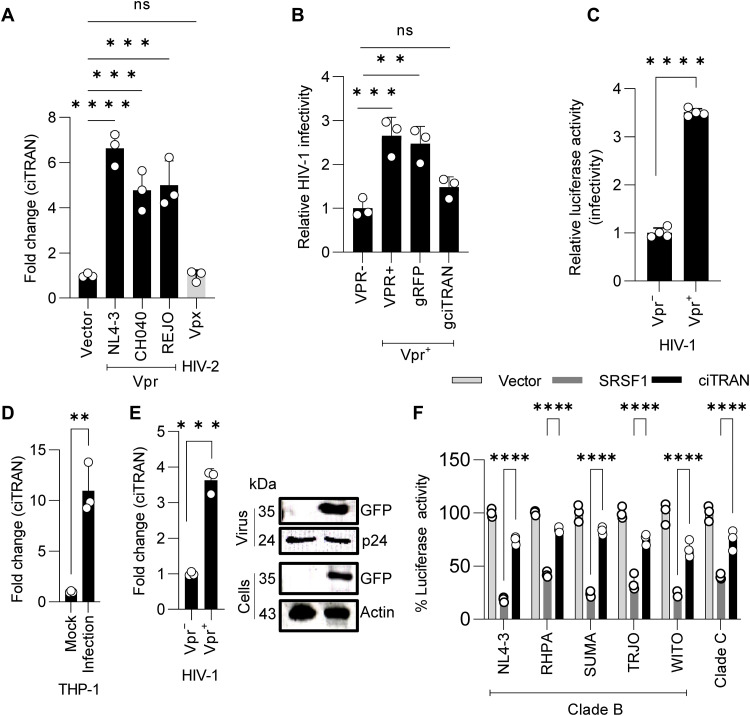
ciTRAN induction by Vpr is conserved and is not cell-specific. (**A**) ciTRAN induction in JTAg T cells by expression of HIV-1 Vpr alleles and Vpx from HIV-2. GAPDH served as a reference for normalization; *n* = 3; ±SD. All the Vpr alleles were ectopically expressed through a pcDNA backbone (plasmids described in the Supplementary Materials). (**B**) Effect of ciTRAN knockdown on HIV-1 Vpr(+/−) Luc infectivity in JTAg cells. ciTRAN knockdown JTAg T cells made using CRISPR-CasRx were challenged with HIV-1 Luc with and without Vpr. Infectivity was scored as relative luciferase units normalized to protein quantified by Bradford method from cell lysate. (**C**) HIV-1 Vpr(+/−) Luc infectivity scored as relative luciferase units from THP-1 monocytes. Normalized to total protein quantified by Bradford method; *n* = 4; ±SD. (**D**) ciTRAN levels in THP-1 monocytes infected with HIV-1–zsGreen; *n* = 3; ±SD. (**E**) ciTRAN levels induced by virion-encapsidated Vpr in THP-1 monocyte cells (left) and Vpr from viruses and from target THP-1 cells (right). HIV-1 Luc (NL4-3 E^−^R^−^ Luc) was produced from HEK293T in the presence of empty vector pEGFP or pEGFP-C2-Vpr. GFP-tagged Vpr expression was confirmed by immunoblotting using an anti-GFP antibody. (**F**) Reporter activity, from HEK293T, driven by LTRs from TFVs and different clades of HIV-1 under ciTRAN and SRSF1 ectopic expression conditions. *n* = 3; ±SD.

We next investigated whether Vpr-mediated ciTRAN induction is a conserved feature. Transfection of pCDNA encoding representative Vpr alleles from NL4-3, transmitted founder viruses (TFVs) CH040 and REJO indicated a conserved feature associated with Vpr alleles. In contrast, Vpx from HIV-2 could not induce ciTRAN expression (Fig. 7A) under these experimental conditions. Vpr interacts with various proteins in host cells and affects important processes such as DNA damage response, cell cycle arrest, replication stalling, and homologous recombination (HR) repression and targets host proteins via proteasomal pathway by recruiting them to DDB1 And CUL4 Associated Factor 1 (DCAF1) (*35*–*39*). We aimed to determine whether activities associated with Vpr, i.e., cell cycle arrest, its ability to engage DCAF1, and modulation of DNA damage response, are responsible for the induction of ciTRAN. To investigate this, we generated different mutants of Vpr (fig. S9F) and expressed them in JTAg cells by transfection and measured the levels of ciTRAN. In these experimental conditions, we observed that a Q65R mutant that was defective in all the reported activities also failed to induce ciTRAN, while S79A and R80A (defective in cell cycle arrest) modestly induced the ciTRAN levels, although not as effectively as the wild-type Vpr (fig. S9G).

Vpr-sufficient HIV-1 is associated with higher virion infectivity in target cells compared to HIV-1 deficient in Vpr (*40*). Our results implicate that ciTRAN induction by Vpr can contribute to infectivity enhancement (Fig. 7B and fig. S9H). In agreement with previous reports (*41*, *42*), Vpr could also enhance viral replication in macrophages (Fig. 7C), and this enhancement of infectivity correlated with Vpr’s virion encapsidation. Therefore, we next examined if virion-packaged Vpr can also induce target cell levels of ciTRAN. THP-1 monocytes challenged with HIV-1 Luc showed more than 10-fold induction of ciTRAN, and virion-encapsidated Vpr (during the production, Vpr is packaged through expression in trans using pEGFP-C2) was sufficient to induce the circRNA (Fig. 7, D and E). Furthermore, additional assessments of ciTRAN induction by virion-encapsidated Vpr using THP-1 as target cells at different multiplicities of infection (MOIs) revealed corresponding dose-dependent ciTRAN induction (fig. S9I). Together, these results revealed that HIV-1 Vpr is required and sufficient to induce the expression of ciTRAN in monocytes and T cells.

### ciTRAN promotes transcription from LTRs of TFVs

As different Vpr alleles were able to induce ciTRAN, we further checked whether the molecular mechanism of HIV-1 transcriptional regulation by ciTRAN is conserved across different HIV-1 isolates. We found a well-preserved SRSF1 binding site on LTR from various HIV-1 isolates, including transmitted founder ones (fig. S10A), reinforcing the importance of the ciTRAN-SRSF1 interplay in regulating viral transcription in this context. To experimentally confirm the effects on transcription and its relevance for field isolates, we checked if the ciTRAN-SRSF1 axis can modulate transcription from LTRs of TFVs and other clades. Toward this, we constructed LTR reporters that expressed luciferase cDNA downstream of LTRs (fig. S10B). Luciferase assays were performed from HEK293T cells transfected with LTR minigene reporter along with Tat and SRSF1 expressors and corresponding empty vectors. SRSF1 ectopic expression phenocopied effects of ciTRAN down-regulation for all the LTRs tested in a manner consistent with its inhibitory effects on LTR-mediated transcription. Notably, this inhibition by SRSF1 was invariably counteracted by ciTRAN expressed from a synthetic construct [pCDNA 3.1(+) circMini ciTRAN] (Fig. 7F), further implying a competing scenario among SRSF1 and the circRNA. Together, these experiments underscore the importance of the ciTRAN-SRSF1 axis in regulating HIV-1 transcription for the field isolates.

SRSF1 expression remained unaltered in the infection (fig. S10C), signifying that a constitutively expressed protein is a bottleneck for HIV-1 transmission. Our findings revealed that ciTRAN can modulate HIV-1 transcription and that the ciTRAN-SRSF1 axis plays an essential role in this process. Plausibly, Vpr antagonizes a constitutively expressed SRSF1 by inducing ciTRAN expression that then excludes the host inhibitor from the viral transcription complex.

## DISCUSSION

By substantially reducing the linear RNA content, we demonstrated the utility of circDR-seq in detecting circRNA BSJs despite the challenges posed by low expression levels and the overrepresentation of viral reads in HIV-infected cells (*19*, *43*). Our efforts may help expand the utility of nanopore DRS for capturing not only circRNAs in the native form but also associated modifications. Moreover, although DRS results in low-read numbers (1 to 2 million reads per flow cell) compared to hundreds of millions of reads that can be obtained using cDNA-based Illumina runs, enrichment using successive linear RNA removal steps could, to a great extent, circumvent the need to obtain a higher number of reads. Our multistep purification procedure and BSJ detection method were validated using experimental validations, various databases, simulated datasets, and comparative assessments with other protocols, showing its reliability in detecting bonafide circRNAs. In addition, circDR-seq enabled the detection of BSJs from circRNAs of a wide range of sizes (ranging from as small as 100 bases to more than 5000 bases). Notably, the direct RNA evidence for unannotated BSJs and their confirmation by gold standards such as Sanger sequencing further highlights the potential of circDR-seq in cataloging previously unannotated circRNAs and understanding their role in other pathophysiologies.

The identification of circRNA function in HIV-1 infection has provided a fresh insight into the molecular mechanisms of HIV-1 transcriptional regulation, and it required cell lines of distinct tissue origins, primary cells, and RNA manipulation tools (such as CRISPR-CasRx) to ensure the validity. However, we acknowledge the limitations of CRISPR-mediated depletion of circRNAs, which only achieved up to 50% knockdown in some cases. Thus, methods that allow obtaining higher knockdown efficiency could reveal the function of additional candidate circRNAs in HIV-1 infection.

Our experiments established a role for ciTRAN (annotated as hsa_circ_0001445) in modulating HIV-1 transcription, possibly via determining the availability of SRSF1 near the HIV-1 transcribing locus. SRSF1 can out-compete Tat binding in the early phase of viral transcription due to the low level of Tat, thereby repressing the HIV-1 transcription (*34*). Our results suggest that the constitutively expressed SRSF1 can be titrated out by ciTRAN, which the viral accessory protein Vpr can induce. These results are in agreement with previous observations from U87 glioma cells, where this interaction was parallelly reported (*44*, *45*). More recently, it has been shown that the GAUGAA motif (on circSMARCA5/ciTRAN) is recognized by the SRSF1 protein (*46*), and this interaction determines the aggressive nature of glioblastoma multiforme. We determined molecular features that SRSF1 requires to bind to ciTRAN, and unexpectedly, we found that the same domain that recognizes the TAR region also binds to the circRNA. While titration of abundant proteins by RNA was thought to be stoichiometrically unlikely, several reports have now established that the bulk of these RNA binding proteins (RBPs) act and function in a liquid-liquid phase separation (LLPs) manner (*47*) and that these condensates are initiated by a relatively small amount of RNAs. RNA multimerization can induce the formation of biomolecular condensates (LLPs, nuclear speckles, P bodies, stress granules, and a variety of other membraneless nuclear compartments) (*48*, *49*). Given the ability of one RNA molecule to sequester more than one protein molecule, a sequestration/sponging model appears to be plausible. One ciTRAN molecule has seven GAUGAA motifs and, in theory, can sequester a correspondingly equal number of SRSF1 proteins, thereby controlling the local availability of these RBPs (*50*). It has been reported that SRSF1 localizes to nuclear speckles (*51*); within these subcellular compartments, HIV-1 replication complexes also operate (*52*). This further suggests that the local availability of RBPs determined by small RNAs in these subcellular compartments can profoundly affect transcription. In our experimental conditions, the copy number can go up to ~35 copies per cell, and by synthetic overexpression constructs, the copy number can go up to ~400 copies per cell for ciTRAN/circSMARCA5 (fig. S10D). By titration experiments, we demonstrated that ciTRAN can intercept SRSF1’s ability to displace Tat from the transcriptional complex and can reinstate Tat association with TAR. These experiments also hint a differential affinity of SRSF1 toward ciTRAN and TAR. The binding of ciTRAN to SRSF1 is also RRM2 dependent, and correspondingly, SRSF1 lacking RRM2 enhances the HIV-1 infectivity. While defining the propensity of RRM2 toward ciTRAN binding by structural studies is in order, we show the feasibility of targeting the virion-induced ciTRAN by generating a synthetic CPP-tagged SRSF1 mimic, which nullifies the effects of circRNA. More work is needed to refine this approach by developing smaller peptides or molecules that can interfere with the transcription from LTR, an event crucial for virion biogenesis and propagation.

HIV-1 accessory protein Vpr is crucial in the early stage of the viral life cycle in myeloid and lymphoid cells (*53*–*56*). Vpr interacts with host cellular proteins, affecting key activities such as cell cycle, host protein degradation by proteasome (*37*, *39*), DNA damage response, nuclear import of pre-integration complex (PIC), apoptosis, antagonizing human silencing hub (HUSH)-mediated epigenetic silencing, and enhancing viral transactivation. While we observe that the Vpr mutants defective in major activities were also unable to induce ciTRAN, further studies are warranted to dissect this molecular cross-talk. Furthermore, the significance of virion-packaged Vpr and its effect on transcription and requirement during the early phase remain incompletely understood (*57*, *58*). Our experimental demonstration that HIV-1 lacking Vpr fails to induce ciTRAN and that virion-encapsidated Vpr is sufficient to induce the circRNA for relieving SRSF1-mediated transcriptional block provides new insights into this long-sought Vpr function necessary for early replication events. The results are also consistent with encapsidated Vpr’s ability to modulate HIV-1 replication in the early stage of infection (*59*, *60*). Vpr could induce SMARCA5 mRNA expression up to fivefold. However, the induction is not reflected in a commensurate rise in SMARCA5 protein levels. One possible explanation for this is the formation of R loops with the parental gene. As recently reported (*61*), these R loops help to produce a shortened form of SMARCA5 transcript, which results in the synthesis of an unstable protein. Vpr is under selective pressure during natural infection, and there is a high degree of conservation across different HIV-1 clades (*62*). Our results suggest that ciTRAN induction occurs in patients irrespective of their antiretroviral therapy (ART) status, with 13 of 15 patients being on ART. This may be attributed to detectable Vpr levels despite ART (*63*) and to the ability of the viral accessory protein to cross the plasma membrane to carry out its function. We found that different HIV-1 Vpr alleles, but not HIV-2 Vpx, could induce ciTRAN, highlighting an HIV-1–specific molecular interplay in modulating circRNA levels to promote viral transcription. Overall, our study offers new insights into HIV-1 transcriptional regulation and highlights the role of host-encoded circRNA during infection.

## MATERIALS AND METHODS

### Cell culture

Jurkat E6.1 [American Type Culture Collection (ATCC)], THP-1 (ATCC), and JTAg (*64*) cell lines were cultured in Gibco RPMI supplemented with 10% performance-tested fetal bovine serum (FBS). PBMCs and CD4^+^ primary T cells were first activated using phytohemagglutinin (PHA; 5 μg/ml) and interleukin-2 (IL-2) (50 IU) and maintained in RPMI. Primary cells after activation were expanded in IL-2 containing RPMI. HEK293T [European Collection of Authenticated Cell Cultures (ECACC)], TZM-GFP (*64*) cells were maintained in 10% FBS containing Dulbecco’s modified Eagle’s medium (DMEM) with 2 mM l-glutamine. The cells were maintained in humidified 5% CO_2_ incubator at 37°C. Cell monolayers were grown at a split ratio of 1:10 by treating with trypsin (0.25%) and 1 mM EDTA (Thermo Fisher Scientific, USA). The list of reagents, sources, and softwares used is provided in Supplementary Table.

### Virus production and infection

For target cell infection, the virus was produced from HEK293T producer cells using the calcium phosphate method by cotransfecting 7 μg of NLBN zsGreen Env defective and Nef defective (HIV-1–zsGreen reporter) and 1 μg of pMD2.G encoding vesicular stomatitis virus G (VSVG) glycoprotein and was limited to single-cycle replication. NLBN zsGreen was a gift from M. Pizzato, which is a derivative of NL4-3 Env Nef^−^ described by Pizzato *et al.* (*65*) and Rosa *et al.* (*64*), where env region is deleted, and zsGreen is cloned in place of Nef. The virus-containing culture supernatant was collected after 48 hours after transfection, centrifuged at 300*g* for 5 min, and then filtered using a syringe filter of 0.22 μm. The virus was quantified using SGPERT assay (*66*), and the MOI was calculated by infecting HEK293T cells. Jurkat E6.1 cells were infected at MOI = 2, whereas primary CD4^+^ T cells and PBMCs cells were infected at MOI = 5 or as indicated. For nanopore sequencing, Jurkat E6.1 T cells were infected in a T75 flask (Eppendorf, Germany) for 48 hours and processed for RNA isolation. The infectivity was quantified using flow cytometry (FACSAria III) by acquiring zsGreen-positive cells.

For virus production from JTAg cells, either 8 μg of NL4-3 Env^−^ R^−^ Luc or NL4-3 Env^−^ R^+^ Luc was electroporated along with 2 μg of VSV glycoprotein-encoding plasmid (pMD2.G). Cells in the exponential growth phase were harvested (10^7^ cells per sample) at 300*g* for 5 min. Cells were washed using phosphate-buffered saline [PBS (1×), pH 7.4] to remove cell debris and residual serum and were resuspended in warm Opti-MEM (200 μl per sample). Next, the samples (cells with plasmid) were added to a 2-mm-gap electroporation cuvette (Bio-Rad, USA). The sample was pulsed at 140 V and 1000 μF with an exponential decay on a Bio-Rad Gene Pulser Xcell module. Warm RPMI (~600 μl) with 20% FBS was then added to the electroporated cells, which were then transferred to a six-well plate containing prewarmed 10% FBS containing RPMI. After 48 hours, the virus supernatant was collected by removing cells using centrifugation, filtered, and stored for further use.

### RNA isolation, cDNA synthesis, and qPCR

For RNA isolation, cells were lysed using TRIzol (Invitrogen, Thermo Fisher Scientific, USA) and phase-separated using chloroform. The aqueous phase containing RNA was processed using an RNA clean and concentrator kit (Zymo research). Samples were treated using deoxyribonuclease (DNase) I to remove any possible DNA contamination. RNA concentration was determined using a Qubit 4.0 fluorometer with a Qubit HS RNA kit (Thermo Fisher Scientific). cDNA first-strand synthesis was achieved by either random hexamers (Thermo Fisher Scientific, catalog no. SO142) or oligo(dT) primers (catalog no. SO132) using SuperScript III (Invitrogen, Thermo Fisher Scientific, USA, catalog no. 18080044). For expression quantification, SYBR Green I (Invitrogen, catalog no. S7585)–based qPCR was performed, and data were represented as log_2_ fold change.

### circRNA enrichment and Nanopore library preparation

For nanopore sequencing, linear RNAs were depleted by various steps to enrich circRNAs. Precisely, linear RNAs were depleted by ribodepletion, polyadenylation, and poly(A) RNA removal, followed by RNase R treatment. This method markedly depleted rRNA, poly(A) RNA, and nonpolyadenylated RNAs, including highly structured RNAs, and provided a highly pure circRNA fraction. Purified circRNA fractions were then fragmented using NEBNext Magnesium RNA fragmentation module and polyadenylated and processed for nanopore DRS protocol. A detailed description of this method is available in the Supplementary Materials.

### RNAse R treatment of the RNA

For degradation of the linear RNA pool, 1 μg of RNA was incubated with 1 U of RNase R at 37°C for 30 min. RNase R was then inactivated at 90°C for 10 min. RNA was then eluted using a Zymo clean and concentrator kit and used for RT-qPCR to quantify respective RNAs.

### Nanopore sequencing and data analysis

The DRS libraries were prepared according to the ONT protocol SQK-RNA002, and RNA sequencing was performed using the MinION platform with a FLO-MIN106 flow cell. The MinKNOW (v3.2.6) interface was used, and data were basecalled using Guppy (v3.2.8), keeping high-accuracy basecalling. The filtered data were mapped to the human genome (hg38) using a Burrows-Wheeler Aligner-Maximal Exact matches(BWA-MEM) aligner and Samtools. For capturing the circRNA from the DRS nanopore reads, we designed a virtual BSJ library of all circRNA present in the circBase (*24*) and circAtlas (*67*) databases. The circRNA sequences were downloaded from circBase (hg19) and circAtlas (hg38) database, respectively. FASTA sequences were converted into back-splice FASTA sequences using the circDR-seq custom script. Then, these back-splice sequences are shortened to 100 bp, 50-bp upstream, and 50-bp downstream to the BSJ. This shortening was done mainly to identify the back-splice read in the nanopore reads.

Further, using pblat (*68*), back-splice sequences were aligned to the sequencing reads (generated in this study). pblat is able to map the sequences across the reads, but when it encounters a back-splicing junction, it gets split into two segments that are individually mapped to the different regions of the same gene. Two segments of a read that match upstream and downstream of a splice site signify a BSJ and, therefore, can be considered a circRNA. To qualify as a circRNA, the Blat score has to reach 60 and should be appropriately aligned 40 bp across the junction (20 upstream and 20 downstream).

### Simulation circRNA reads

To generate simulated nanopore circRNA sequencing datasets, we used an existing tool NanoSim (*69*). Toward this, we downloaded circBase fasta sequences and selected the top 3000 circRNA sequences for generating simulated reads and converted these 3000 circRNA linear fasta sequences into back-splice fasta sequences. Subsequently, simulated reads were generated using the “genome mode” provided in NanoSim. In total, 12 million reads were simulated, which resulted in aligned simulated reads fasta file and unaligned simulated reads fasta file. Only the aligned simulated reads fasta file was used for downstream analysis, consisting of back-spliced reads. The performance assessment of circDR-seq was done by precision and recall rates of circRNA identification, and the overall performance was evaluated by the F1 score using the following equation

$$F1\;score=2\times \frac{\left(Precision\times Recall\right)}{\left(Precision+Recall\right)}$$

### CasRx-based circRNA knockdown and infectivity assay

For circRNA knockdown, CasRx- and gRNA-expressing plasmids (1:2 ratio) were cotransfected into JTAg cells by electroporation. Cells in the exponential growth phase were harvested (10^7^ cells per sample) at 300*g* for 5 min. Cells were washed using PBS (1×, pH 7.4) to remove cell debris and residual serum and were resuspended in warm Opti-MEM (200 ml per sample). A total of 8 μg of total plasmid [2 μg of CasRx-expressing and 4 μg of gRNA-expressing vectors and 2 μg of pCDNA 3.1 BS(−)] was then mixed into the suspended cells. Next, the samples were added to a 2-mm-gap electroporation cuvette (Bio-Rad, USA). The sample was pulsed at 140 V and 1000 μF with an exponential decay on a Bio-Rad Gene Pulser Xcell module as described previously (*70*). Warm RPMI (~600 ml) with 20% FBS was immediately added to the electroporated cells, which were then transferred to a sixwell plate containing prewarmed 10% FBS containing RPMI. After 48 hours, cells were spun down at 300*g* for 5 min, resuspended in fresh medium, and selected using blasticidin for 4 days. Cells were divided into two parts; one part was used to analyze the knockdown efficiency of the circRNAs, and the other part, having the same number of cells, was processed for HIV-1 infectivity analysis using the HIV-1 Luc virus. For HIV-1 infectivity, luciferase-containing virus (produced from single-cycle NL4-3 Luc (defective in Env, Vpr, and Nef) was used. After 48 hours, cells were collected and processed for luciferase assay.

### m6A circRNA pulldown and analysis

For m6A pulldown, the total RNA (10 μg) was subjected to RNase R digestion, followed by cleanup using an RNA clean and concentrator kit (Zymo). Linear RNA-depleted fraction was then fragmented using the magnesium RNA fragmentation module using the manufacturer’s protocol. Purified RNA was then pulled down using m6A-specific antibody using an EpiMark N6-methyladenosine enrichment kit (NEB). RNA was converted into cDNA using random hexamer primers and RevertAid RT (Thermo Fisher Scientific). Selected candidates were then analyzed by RT-qPCR using specific primers. Data were normalized to input with IgG and m6A fractions.

### Measurement of circRNA copy number

To generate a standard curve for qPCR, a purified known DNA template was serially diluted. The copy number of the diluted DNA template was calculated using the DNA/RNA copy number calculator available at the following website: http://sciprim.com/html/copyNumb.v2.0.html. To measure the ciTRAN copy per cell, the total RNA was extracted from 1 million Jurkat E6.1 cells and then diluted ten-fold. The splint-based probe ligation technique followed by qPCR-based ligation was used to quantify the copy number of ciTRAN from the standard curve. In addition, the copy number of overexpressed ciTRAN was also measured using the same method described above.

### Luciferase assay

Luciferase assay was performed in 96-well plates to analyze viral infectivity upon circRNA knockdown. Equal numbers of cells were lysed in 96-well plates using 100 μl of lysis buffer [1% Triton X-100 (Sigma-Aldrich), 25 mM tricine (VWR), 15 mM potassium phosphate (at pH 7.8), 15 mM MgSO_4_, 4 mM EGTA, and 1 mM dithiothreitol (DTT)] for 20 min at room temperature. Luminescence was measured using SpectraMax i3X plate reader (Molecular Devices, USA) by injecting 50 μl of substrate buffer [lysis buffer + 1 mM DTT (VWR Scientific), 1 mM adenosine 5′-triphosphate (ATP) (Sigma-Aldrich), and 0.2 mM d-luciferin (Cayman chemical, USA)] in 50 μl of cell lysate in 96-well white plates (SPL life sciences, catalog no. 30196). Data were normalized to Bradford readings of the cell lysate in case of viral infectivity based on VSVG pseudotyped HIV-1 (NL4-3 Env^−^ R^−^ Luc or NL4-3 Env^−^ R^+^ Luc). For the LTR-Luc experiment, LTR from NL4-3 and from different TFVs was cloned into PGL3-Luc basic vector (Promega). For analyzing the effect of ciTRAN knockdown on different HIV LTRs, LTR-Luc constructs along with pCasRx and pCDNA Tat were transfected into HEK293T with nontargeting gRNA(gRFP) or ciTRAN gRNA. After 48 hours, a luciferase assay was performed to understand the effect of ciTRAN knockdown on LTR activity. Data were normalized to Renilla luciferase cotransfected (10 ng) in every condition. Similarly, for analyzing the effect of SRSF1 and ciTRAN modulation on different LTRs, PGL3-Luc or PGL3-LTR-Luc along with vector or pCDNA Tat was transfected in HEK293T along with SRSF1 and pCDNA 3.1(+) circMini ciTRAN. After 48 hours, a luciferase assay was performed to analyze the effect of SRSF1 and ciTRAN levels on LTR activity.

### Subcellular fractionation

Subcellular fractionation was done as described previously (*71*). For the cytoplasmic fraction, 1 million cells were collected at 500*g* at 4°C for 5 min. The supernatant was removed, and the cell pellet was stored on ice. Cells were resuspended gently in 380 μl of ice-cold hypotonic lysis buffer (HLB) supplemented with RNase inhibitor. Cells were incubated for 10 min, vortexed briefly, and then centrifuged at 1000*g* for 3 min at 4°C. The supernatant containing cytoplasmic fraction was transferred to a new tube, and the pellet was stored on ice. The supernatant was mixed with 1 ml of RNA precipitation solution and stored at −20°C for at least 1 hour. After incubation, the sample was centrifuged at 18,000*g* at 4°C for 15 min, and the pellet was processed for RNA isolation using TRIzol and DNA isolation using the phenol-chloroform method. The nuclear fraction containing the pellet was washed thrice with 1 ml of ice-cold HLB and centrifuged at 300*g* at 4°C for 2 min. Pellet was processed for DNA isolation using the phenol-chloroform method.

### Estimation of HIV-1 reverse transcription, integration, and transcription upon ciTRAN knockdown

For reverse transcription and integration analysis, DNA was isolated from the cytosolic fraction and nuclear fraction, respectively, at different time points and was analyzed using qPCR using the specific primers forward 5′-TCTGGCTAACTAGGGAACCCA-3′ and reverse 5′-CTGACTAAAAGGGTCTGAGG-3′ in case of reverse transcription analysis. HIV-1 integration was quantified with a two-step Alu-gag PCR where, in the first step, genomic DNA with Alu forward 5′-GCCTCCCAAAGTGCTGGGATTACAG-3′ and *gag* reverse 5′- TACCATTTGCCCCTGGAGGTT-3′ was preamplified. The amplicon from this PCR was used as a template for the second round of qPCR using HIV-1 *gag*-specific primers (forward 5′-TCTGGCTAACTAGGGAACCCA-3′ and reverse 5′-CTGACTAAAAGGGTCTGAGG-3′). HIV-1 RNA (*gag*) abundance from cytoplasm as a measure of HIV-1 transcription was quantified at different time points using forward 5′-TTGTACTGAGAGACAGGCT-3′ and reverse 5′-ACCTGAAGCTCTCTTCTGG-3′. The RNA was isolated using TRIzol/chloroform method, and the cDNA synthesis was performed using the random hexamer primer.

### Nuclear run-on assay

A nuclear run-on assay was performed as described previously (*72*). For nuclei isolation, cells were harvested using ice-cold hypotonic solution [150 mM KCl (Sigma-Aldrich), 4 mM MgOAc (Sigma-Aldrich), and 10 mM tris-HCl (Sigma-Aldrich) (pH 7.4)] and were pelleted by centrifugation 300*g* for 5 min. Next, the cell pellet was resuspended in lysis buffer [150 mM KCl, 4 mM MgOAc, 10 mM tris-HCl (pH 7.4), and 0.5% NP-40]. The nuclear run-on mixture [10 mM ATP, cytidine 5′-triphosphate, guanosine 5′-triphosphate, 5-bromouridine-5′-triphosphate, and the crude nuclei] was incubated at 28°C for 5 min in the presence of RNase inhibitor (Invitrogen). The RNA was then isolated by TRIzol reagent (Invitrogen) as per the manufacturer’s instructions, and the DNA was eliminated by DNase I (Takara) treatment. Nascent transcripts were then immunoprecipitated by anti–5-bromo-2′-deoxyuridine antibody and converted to cDNA for qPCR analysis. qPCR analysis for *gag* was done using forward 5′-TTGTACTGAGAGACAGGCT-3′ and reverse 5′-ACCTGAAGCTCTCTTCTGG-3′ primers.

### CRISPR-assisted RNA-protein interaction detection method

We generated gRNA targeting the back-splicing junction of ciTRAN and cloned it into an empty gRNA-expressing vector (Addgene #109054). The gRNA sequences were designed by keeping GC% = 40 to 60% and filtered for off-targets. A total of 5 μg of gRNA-expressing plasmid (ciTRAN) and 5 μg of Biotin ligase from *Bacillus subtilis* (BASU)-dCasRx constructs were cotransfected by electroporation. Before collection, cells were washed thrice with ice-cold PBS and lysed using 500 ml of lysis buffer [50 mM tris-HCl (pH 7.4), 150 mM NaCl, 0.5% Triton X-100, and 1 mM EDTA supplemented with fresh EDTA-free protease inhibitor cocktail (Roche)] at 4°C for 15 min with rotation. The lysate was spun down at 16,000*g* for 15 min at 4°C. The supernatant was quantified using Bradford (B1696, Sigma-Aldrich) and normalized for protein concentration. Biotinylated proteins were enriched with MyOne T1 streptavidin beads (Thermo Fisher Scientific) after 2 hours of incubation at 4°C with rotation, and three washes were performed with 1 ml of ice-cold lysis buffer. Proteins were eluted from the beads using 2× SDS loading buffer by incubating for 10 min at 95°C and subjected to SDS-PAGE. Following washing with MS grade water, silver staining was performed. The visible differential protein band was excised from the gel and processed for LC-MS analysis. Obtained spectra were run on a Mascot server to identify the target proteins.

### Immunoblotting

For immunoblotting, samples were lysed using radioimmunoprecipitation assay (RIPA) lysis buffer and mixed with Laemmli buffer. Samples were either run on 8 or 12% tricine gels depending upon protein size. Next, gels were electroblotted on the polyvinylidene difluoride membrane (Immobilon-FL, Merck Millipore). The membrane was blocked using a commercial blocking buffer (Bio-Rad) for 5 min, followed by primary and secondary antibody incubations for 1 hour each at room temperature. Post-antibody incubation membrane was washed thrice (5 min per wash) using Tween 20 containing 1× tris-Buffered saline. Detection of β-actin, p24, SRSF1, and GFP was carried out using anti–β-actin, mouse anti-p24, anti-SRSF1, and anti-GFP. Details of antibodies are provided in Supplementary Table.

### Photoactivatable ribonucleoside-enhanced crosslinking and immunoprecipitation

PAR-CLIP was performed as described previously (*73*). The E6.1 cell line was expanded 2 days before the infection, and the old medium was replaced with the fresh medium before infection. Next, cells were collected and divided into two flasks, cells in one flask were challenged with HIV-1 particles at MOI 5, and the second flask was challenged with a mock referring to it as control. In the next step, the virus-containing medium was replaced after 6 hours with a fresh medium. Cells were incubated with 100 mM 4-thiouridine for 16 hours. Following incubation, cells were collected using centrifugation and washed twice with ice-cold PBS. After washing, cells were resuspended in ice-cold PBS and crosslinked thrice with a 1-min gap using a 365-nm based UVP crosslinker at 0.2 J/cm^2^. Following the crosslinking step, cells were collected and either stored in −80 until further use or directly processed for the pull-down. For the pull-down experiment, protein G beads were initially incubated with the SRSF1 (5 μg) antibody or IgG at room temperature for 40 min. After lysing a sample with NP-40 (Sigma-Aldrich)–based lysis buffer, the lysate was cleared using a centrifuge, and the supernatant was added to the preoccupied antibody-beads mixture. The mixture was then incubated at 4°C overnight. Post-incubation beads were washed with high-salt buffers and lastly dissolved in proteinase K digestion buffer for proteinase K digestion (Thermo Fisher Scientific). After that reaction was stopped by adding 1 ml of TRIzol, RNA isolation and cleanup were performed using a Zymo clean and concentrator kit. Isolated RNA was then processed for cDNA synthesis. Similarly, for CPP-RRM2-HA, PAR-CLIP was performed on the basis of HA antibody.

### Effect of SRSF1 on host genes splicing

We used RNA sequencing data GSE163025 obtained from a previous investigation of SRSF1 wild-type and knockdown cells. Subsequently, we used a differential splicing pattern analysis to identify genes that were differentially spliced between these two conditions. We further analyzed the differentially expressed genes (SMARCAD1, NAV1, GAB1, HDAC7, TERF1, and MAPK11) to identify those that were potentially relevant to HIV-1 infection. We designed exon-specific primers for individual genes and performed splicing analysis using PCR after cDNA synthesis using RevertAid reverse transcriptase and oligo(dT) primers during empty vector [pCDNA 3.1(+) circMini] and ciTRAN expression. Similarly, we checked HIV-1 alternative splicing during ciTRAN overexpression from a 2-kb multispliced RNA fragment. Subsequently, HIV-1 unspliced, singly spliced, and multispliced HIV-1 RNAs were also checked during ciTRAN knockdown using specific primers. Primer sequences are available in auxiliary data table S1.

### Chromatin immunoprecipitation

For ChIP, 10 million cells were cross-linked for 10 min at room temperature with 1% formaldehyde and then quenched with 0.125 M glycine for 5 min at room temperature. Cells were washed three times in cold PBS before being resuspended in a buffer containing 50 mM Hepes-KOH (pH 7.5), 140 mM NaCl, 1 mM EDTA, 10% glycerol, 0.5% NP-40, 0.25% Triton X-100, and protease inhibitors. Nuclei were pelleted at 800*g* for 5 min at 4°C and resuspended in a buffer containing 10 mM tris-HCl (pH 8.0), 200 mM NaCl, 1 mM EDTA, 0.5 mM EGTA, and protease inhibitors and then incubated on ice for 10 min. The nuclei were collected and resuspended in a sonication buffer containing 10 mM tris-HCl (pH 8.0), 100 mM NaCl, 1 mM EDTA, 0.5% EGTA, 0.1% sodium deoxycholate, and 0.5% *N*-lauryl sarcosine, as well as protease inhibitors.

DNA was sonicated using a probe sonicator for 15 cycles of 30 s and 45-s off to obtain an average fragment length of 300 to 700 bp. Samples were centrifuged at 16,000*g* for 10 min at 4°C after being treated with 1% Triton X-100. During each sonication cycle, an aliquot of sonicated DNA was reverse-crosslinked and run on a 1% agarose gel to confirm the fragment size.

Chromatin (25 μg) was immunoprecipitated by adding the antibody of interest (anti-SRSF1, anti-RNAPII, and anti-Tat), followed by overnight incubation at 4°C. After overnight incubation, 30 μl of protein G beads (Invitrogen) were added and incubated for 1 hour at 4°C. Beads were washed sequentially for 3 min each in low salt [20 mM tris-HCl (pH 8.0), 150 mM NaCl, 2 mM EDTA, 0.1% SDS, and 1% Triton X-100], high salt [20 mM tris-HCl (pH 8.0), 500 mM NaCl, 2 mM EDTA, 0.1% SDS, and 1% Triton X-100], LiCl buffer [10 mM tris-HCl (pH 8.0), 0.25 M LiCl, 1% NP-40, and 1% sodium deoxycholate] and Tris EDTA (TE) buffer. Beads were eluted in 150 μl of elution buffer [50 mM tris-HCl (pH 8.0), 10 mM EDTA, 1% SDS, and 50 mM NaHCO_3_] and treated with 1 μl of RNase A (1 mg ml^−1^; Ambion) at 37°C for 30 min. Cross-linking was reversed, and proteins were degraded by adding 1 μl of proteinase K and incubated at 65°C for 4 h. Eluted DNA was purified and used for qPCR analysis.

### Virion incorporation of Vpr

Virus incorporation of protein assay was done as described previously (*70*). Virus particles (HIV-1 Luc) were produced by transfecting HEK293T cells using calcium phosphate transfection reagent in a 10-cm plate, NL4-3 Env^−^ R^−^ Luc, pMD2.G (VSVG) along with 2 μg of Vpr expressing construct (pEGFP-C2-Vpr), or vector alone. After 12- to 15-hour transfection, the medium was replaced with 2% FBS containing DMEM. After 48 hours, the virus-containing supernatant was collected at 500*g* for 5 min to exclude any cell debris. Next, the virus-containing supernatant was filtered using a 0.22-mm syringe filter. The suspension was overlaid on a 20% sucrose (prepared in 1× PBS) cushion and concentrated at ~110,000*g* for 2 hours at 4°C using a Beckman-Coulter ultracentrifuge. After the spin, the supernatant was discarded, and the pellet was suspended in Laemmli buffer containing 50 mM tris(2-carboxyethyl) phosphine hydrochloride.

### THP-1 infection at different MOIs

Virus production in the presence or absence of Vpr was done from HEK293T cells by transfecting NL4-3 E^−^R^−^ Luc/NL4-3 E^−^R^−^ Luc with pEGFP-C2-Vpr along with VSV envelope. The virus MOI was calculated by infecting TZM-GFP cells. THP-1 cells were infected at various MOI for 6 hours, and the medium was changed after that. Cells were grown for 24 hours, and ciTRAN levels were calculated using RT-qPCR.

### Expression of Vpr, Vpr mutants, and immunoblotting

Vpr mutants were generated using PCR mutagenesis and cloned in pcDNA 3.1(−) along with HA. All the mutants were electroporated in JTAg cells along with wild-type Vpr and checked for expression by immunoblotting using an anti-HA. ciTRAN level was estimated after 48 hours of transfection.

For Vpr expression and SRSF1 and SMARCA5 level estimation, pEGFP-C2-Vpr was electroporated in JTAg cells, and cells were processed for Western blot and RNA isolation using RIPA lysis buffer and TRIzol, respectively. mRNA level quantification was done using specific primers after converting RNA into cDNA using oligo(dT) primers.

### ChIP sequencing data analysis

We conducted an analysis of publicly available ChIPsequencing datasets for SRSF1 and Pol-II using ChIP Atlas (https://chip-atlas.org/). The SRSF1 datasets we examined include SRX3322503, SRX3322504, SRX4708013, SRX3322820, SRX3322821, and SRX4708061. For Pol-II, we analyzed SRX14894051, SRX14894052, and SRX14894053. From these datasets, we shortlisted Dicer1 and NFATC3 as positive candidates for SRSF1 ChIP, while for Pol-II, it was challenging to identify a region where Pol-II was not bound, so we could not select an appropriate negative control. However, we used SRSF7 as a positive control for Pol-II in our experiments.

### Knockdown cells generation

SRSF1 and QKI knockdown JTAG cells were generated using pLKO.1 mission shRNAs from Sigma-Aldrich. We generated lentiviral particles from HEK293T by cotransfecting respective shRNA encoding plasmids or control shRNA (shLuc or shGFP) along with psPAX2, a packaging plasmid, and pMD2.G, a VSV glycoprotein encoding plasmid using calcium phosphate method. The medium was replaced after 12 to 15 hours after transfection, and lentiviral particles were collected after 48 hours. Lentiviral particles containing medium were centrifuged at 500*g* and filtered using a syringe filter of 0.22 μm. JTAg cells were infected using SRSF1 and QKI shRNA particles and selected using puromycin for 1 week.

### SRSF1 deletion mutants and CPP-RRM constructs

SRSF1 full-length gene was cloned in pCDNA 3.1(−) HA vector using Xba I and BspE I after PCR amplification from cDNA using specific primers. Different mutants of SRSF1, such as RRM1, RRM2, and RS, were then generated on this backbone, keeping HA in the frame using PCR mutagenesis (primers are provided in the auxiliary data table S1). Cloning was confirmed by Sanger sequencing, and the expression of each construct was confirmed using anti-HA immunoblotting.

The secretory CPP-tagged RRM2 mimic was cloned in-house. The RRM2 domain alone from SRSF1 was cloned into an Erythropoietin (EPO)-derived secretory peptide–CPP sequence containing plasmid, which was previously generated by us. The CPP sequence used is GAYARKAARQARAGVD. The CPP-tagged RRM2 is expressed as an HA tag at the C terminus by cytomegalovirus promoter. For expressing the CPP-RRM2-HA, we transfected 15 μg of plasmid in HEK293T cells (100-mm plates) using the calcium phosphate method. After 12 hours, media was replaced with advanced DMEM (serum-free), and the supernatant was collected twice at 24- and 48-hour time points. The supernatant containing CPP-RRM2-HA peptide was then concentrated overnight using the ammonium-sulfate precipitation method (45% saturation), followed by desalting using different cut-off membranes (30 and 3 kDa, respectively). Final proteins were brought up to 5 ml. To check whether it can be used for HIV-1 infectivity inhibition, HEK293T cells were infected with VSVG-pseudotyped HIV-1, and after 6 hours, cells were washed, and fresh medium was supplemented along with varying dilutions of CPP-RRM2-HA (1:1, 1:10, and 1:100). Infectivity was scored after 48 hours using SpectraMax i3x multimode plate reader. For the Luciferase assay, the plasmids containing Renilla Luciferase (normalization control), LTR-Luciferase, and Tat were cotransfected with CPP-RRM1/2-HA or full-length SRSF1 (positive control), and the luciferase activity was scored at 48 hours after transfection. For immunofluorescence, the CPP-RRM1/2 HA was cotransfected along with pEGFP-N1, and the signal for HA was detected using primary labeled anti–HA-DyLight 650.

### PBMC isolation from peripheral blood

Blood was collected with informed consent from healthy donors in a preservative-free anticoagulant (0.2% final concentration of EDTA) and processed immediately for PBMC isolation. PBMC isolation was done with the help of Histopaque using the manufacturer’s protocol. Precisely, 3 ml of anticoagulated blood was layered on top of an equal amount of Histopaque-1077. Afterward, the sample was carefully centrifuged in a swing bucket rotor, keeping acceleration and brake at the lowest setting at 400*g* for 30 min at room temperature. Following centrifugation, cells from the opaque interface were transferred to a fresh centrifuge tube. Cells were washed twice with isotonic PBS collected upon centrifugation at 250*g* for 10 min. Last, cells were resuspended in the isotonic PBS or RPMI 1640 and processed further for the CD4^+^ cell isolation.

### CD4^+^ cell purification

CD3^+^/CD4^+^ T cells were purified by positive selection using magnetic separation with a CD4^+^ isolation kit (Miltenyi Biotec) from PBMCs as per the manufacturer’s protocol. Purified CD4^+^ T cells were characterized following counter staining with anti–CD4-allophycocyanin antibody (1:200) using flow cytometry (Miltenyi Biotec), and anti–CD3–fluorescein isothiocyanate–labeled antibody. Antibodies were diluted in PBS/1% bovine serum albumin/0.05% NaN_3_ (PBA).

### Primary T cells maintenance

Primary CD4^+^ T cells were grown and maintained in RPMI 1640 supplemented with 10% FBS. For expanding the CD4^+^ T cells, the cells were either activated with PHA (5 μg/ml; Sigma-Aldrich) or CD3/CD28 beads (1:1 bead-to-cells ratio) and recombinant human IL-2 (50 IU/ml). IL-2 was also supplemented to each culture for the maintenance of the cells.

### Patient sample collection and RNA isolation

Whole blood from patients was collected with informed consent in DNA/RNA shield blood collection tubes (Zymo). RNA was isolated from 1 ml of total blood from 15 patient’s samples using TRIzol plus kit method. RNA was then quantified using Nanodrop, and 500 ng of RNA was converted into first-strand cDNA using reverse transcriptase (Invitrogen) with the help of random hexamer primers. The ciTRAN level was estimated using the RT-qPCR method using specific primers, and samples from healthy donors were taken as control. Data were normalized to housekeeping genes and represented as bar graphs.

### Statistical analysis

Statistical analyses were performed using GraphPad Prism 9.0. Experiments were performed at least three times, and data were presented as the means ± SD from technical replicates. The two-tailed Student’s *t* test (paired/unpaired) or one-way analysis of variance (ANOVA) (multiple comparisons) was used to assess the significance between two or more groups. All reported differences were *P < 0.05, **P < 0.01, ***P < 0.001 and ****P < 0.0001 unless otherwise stated.

## Supplementary Materials

### This PDF file includes:

Supplementary Text Figs. S1 to S10 Legends for auxiliary tables S1 to S7 Legends for auxiliary data files S1 and S2 References

### Other Supplementary Material for this manuscript includes the following:

Auxiliary tables S1 to S7

Auxiliary data files S1 to S2
