# Supplementary material for: HIV-1 Vpr induces ciTRAN to prevent transcriptional repression of the provirus
Source: Sci Adv. 2023 Sep 6;9(36):eadh9170. doi: 10.1126/sciadv.adh9170 (PMC10482341; doi:10.1126/sciadv.adh9170)
Supplement: Supplementary file 1 — Supplementary Text Figs. S1 to S10 Legends for auxiliary tables S1 to S7 Legends for auxiliary data files S1 and S2 References [file sciadv.adh9170_sm.pdf]

Science Advances
AAAS

Supplementary Materials for

HIV-1 Vpr induces ciTRAN to prevent transcriptional repression of the provirus

Vipin Bhardwaj *et al.*

Corresponding author: Ajit Chande, ajitg@iiserb.ac.in

Sci. Adv. **9**, eadh9170 (2023)
DOI: 10.1126/sciadv.adh9170

The PDF file includes:

Supplementary Text
Figs. S1 to S10
Legends for auxiliary tables S1 to S7
Legends for auxiliary data files S1 and S2
References

Other Supplementary Material for this manuscript includes the following:

Auxiliary tables S1 to S7
Auxiliary data files S1 and S2

Supplementary Text

A detailed description of the circDR-seq

Ribosomal RNA (rRNA) depletion

We used the NEBNext rRNA depletion kit v2 (NEB #E7405) to deplete the rRNA, which works based on the RNaseH mediated degradation of rRNA bound to probes. For efficient removal, 50 µg RNA was divided into two parts and used in 20 µL rRNA depletion reactions separately, using the manufacturer's protocol. Initially, the RNA probe master mix was prepared by adding 1 µL of NEBNext rRNA depletion solution and 2µL of probe hybridization buffer to the PCR tube containing 12 µL of the total RNA sample. Further, the reaction was mixed thoroughly by pipetting up and down at least ten times. Next, the reaction was immediately transferred to the thermocycler and set up as follows: 95°C for 2 min, 95-22 °C with a ramp rate of 0.1°C/min, and hold at 22°C for 5 min. The sample was spun down briefly and immediately proceeded for the RNaseH digestion. For RNaseH digestion, five microliters master mix containing 2 µL RNaseH, 2µL RNaseH reaction buffer, and 1 µL of nuclease-free water was added to the previous reaction and after mixing incubated sample at 37°C for 30 min. Post incubation, the sample was spun down and kept on ice. Immediately, the sample proceeded next to DNaseI digestion to degrade the rRNA probes. For DNaseI digestion, 30 µL of master mix containing 5 µL DNaseI reaction buffer, 2.5 µL DNaseI, and 22.5 µL nuclease-free water was added to the RNA from the previous step and incubated at 37°C for 30 min, and immediately proceeded to the next for RNA purification.

RNA purification using magnetic beads

RNA purification was done using the standard NEBNext RNA purification module; briefly, 110 µL RNAClean XP beads were added to the RNA sample in the 1.5 mL DNA LoBind tube (Cat#0030108051, Eppendorf) and mixed thoroughly by pipetting up and down 8-10 times and then incubated on ice for 15 min. Next, the tube was transferred to the magnetic rack and incubated until the solution became clear. Further, the supernatant was removed carefully, and the pellet was washed twice using 80% freshly prepared ethanol for 30 seconds while keeping the tube on the rack. After removing the tube from the stand, RNA was eluted from the pellet using 8 µL nuclease-free water and mixed by pipetting 8-10 times. After that sample was incubated further for 2 min, and the tube was transferred back to the magnetic rack, and the clear supernatant was collected in 0.2 mL RNase-free PCR tubes (Axygen). The RNA was supplemented with Ribolock RNase inhibitor (Thermo Fisher Scientific, EO0381) to preserve its integrity and quantified using Qubit 4.0 using RNA quantification HS kit. rRNA depletion was confirmed by RT-qPCR using rRNA-specific primers. Primer sequences are provided in the Data Table S1. RNA clean and concentrator™ -5 (R1016, Zymo Research) was used for RNA clean-up and concentration purposes.

RT-qPCR analysis and validation for rRNA depletion

Purified RNA was further used for cDNA synthesis using SuperScript® III First-Strand Synthesis System (Cat#18080044, Invitrogen) using manufacturer's protocol in a 20 µL reaction containing RNA, 1x first-strand synthesis buffer, dNTPs, and random hexamer primers, DTT and

reverse transcriptase (Thermo Fisher Scientific). The cDNA reaction mixture was then incubated at 25°C for 5 min followed by 60 min at 50°C; the RT enzyme was then inactivated by heating at 70°C for 15 min. After cDNA synthesis, cDNA was diluted 10-fold and used in PCR to confirm rRNA depletion using specific primers (Data Table S1). After the rRNA depletion, RNA was then used for further processing.

Polyadenylation of the linear RNA for depletion
Poly(A) tail was added to remaining linear RNAs after rRNA depletion, including fragmented mRNAs, residual rRNAs, and short 3' overhangs containing snRNAs using E. Coli poly(A) polymerase (Cat#M0276L, NEB). A 20 µL reaction containing RNA, 1x E. coli Poly(A) Polymerase Reaction Buffer, 1mM ATP, Ribolock RNase inhibitor, and 5 U E. Coli poly(A) polymerase was incubated at 37°C for 30 min. Immediately, post-incubation RNA was processed further for purification using an RNA clean and concentrator kit (Zymo Research).

Depletion of poly(A) RNA
Following polyadenylation of the RNA, the sample was processed for poly(A) RNA depletion using NEBNext poly(A) mRNA Magnetic Isolation Module (New England Biolabs). RNA was diluted with nuclease-free water to make a final volume of 50 µL in a 0.2 mL RNase-free PCR tube. In a second PCR tube, 20 µL of NEBNext Magnetic Oligo d(T)25 Beads were aliquoted and washed twice by adding 100 µl of RNA binding buffer and mixed thoroughly by pipetting up and down at least six times. The tube was placed on the magnetic rack until the solution became clear. The supernatant was then discarded, the tube was removed from the magnetic rack, and magnetic beads were resuspended using 50 µL RNA binding buffer and then mixed with 50 µL RNA reaction from tube one. The reaction was mixed thoroughly using a pipette and then incubated on a thermal cycler at 65°C for 5 min and then further held at 4°C for denaturation and binding of poly(A) RNA to the magnetic beads. Once samples reached 4°C, the sample was removed and mixed using a pipette 8-10 times and incubated at room temperature for 5 min. The sample was again mixed and incubated and repeated further for five more minutes. Then the sample was kept on a magnetic stand until it became clear. This time, the supernatant containing the RNA pool of interest was collected carefully and transferred to a fresh 1.5 mL DNA LoBind tube (Eppendorf). The supernatant was subjected to RNA isolation using RNA clean and concentration (Zymo Research).

RNaseR treatment and validation of poly(A) mRNA depletion
Despite efficient removal of rRNA and poly(A) RNAs, including mRNA, possible contamination of small fractions of fragmented RNAs is always possible. To enrich the highly pure fraction of circular RNAs, we treated the sample using RNaseR. RNaseR is quite efficient in degrading the residual linear fraction, provided the sample complexity is low. Therefore, treating samples at this stage using RNaseR will enrich the presence of circular RNAs as it will degrade all the linear fractions efficiently. A 20 µL reaction was set up using RNA which was supplemented with 1x RNaseR buffer and 20U of RNaseR. Ribolock RNase inhibitor (Thermo Fisher Scientific) was also supplied in the reaction to prevent circRNA degradation. The complete reaction was then incubated at 37 °C for 30 min. Post incubation, RNA was isolated from the reaction using RNA clean and concentrator following the manufacturer's (Zymo research) protocol and eluted in 10 µL of nuclease-free water. Isolated RNA was used further for cDNA synthesis using reverse transcriptase (Superscript III) with random hexamer primers. To confirm the depletion of

poly(A) mRNA and validation for circular RNAs enrichment, PCR followed by 2% agarose gel electrophoresis was performed using circular RNAs and GAPDH-specific primers. At this step, obtained RNA is highly enriched in circular RNAs and can also be used further for sequencing studies and library preparation.

Circular RNA fragmentation

Further, we used a NEBNext Magnesium RNA Fragmentation Module (New England Biolabs) to linearize the circular RNA in preparation for RNA polyadenylation for direct RNA sequencing using Nanopore. In order to generate fragments of different sizes, RNA was divided into three parts, and RNA fragmentation was done for 30 sec, 1 min, and 1.5 min, respectively. All three reactions were pooled together, and RNA was concentrated in the same way as described previously. Since fragmentation produces ends with 3' phosphate, the 3' phosphate group was removed and added to the 5' ends using T4 polynucleotide Kinase (New England Biolabs) by first incubating for 30 minutes each without/with ATP to remove and add the phosphate group, respectively.

Polyadenylation of Circular RNA fragments

Fragmented circular RNAs were next polyadenylated using E.coli poly(A)polymerase (New England Biolabs) for 30 minutes. Post polyadenylation RNA was concentrated using RNA clean and concentrator^{TM} (Zymo Research). Polyadenylated RNA was further quantified using Qubit 4.0 using RNA HS kit and proceeded for Nanopore library preparation.

RT adaptor ligation and Reverse transcription

For nanopore library preparation, 9µl of poly(A) RNA, 3.0 µl NEBNext Quick Ligation Reaction Buffer, 0.5 µl RNA CS (RCS), 1.0 µl RT adapter, and 1.5 µl T4 DNA ligase were added in a 0.2 ml thin-walled PCR tube. The reaction was mixed thoroughly using pipetting. The reaction mixture was incubated for 10 minutes. After that, a reverse transcription master mix (9.0 µl nuclease-free water, 2.0 µl 10 mM dNTPs, 8.0 µl 5x first-strand buffer, and 4.0 µl 0.1 M DTT) was added to the RT adapter-ligated RNA reaction and mixed by pipetting. Finally, 2µl of Superscript III reverse transcriptase was added to the reaction, and cDNA first-strand synthesis was performed with thermocycler using the following protocol: one cycle at 50^{0}C for 50 minutes, one cycle at 70 ^{0}C for 10 min for heat inactivation, and finally cool down to 4^{0}C.

RNA cleaning and purification

After first-strand synthesis, the sample was transferred to a clean 1.5 mL DNA LoBind tube (Eppendorf), well-suspended 72 µl of Agencourt RNAClean XP beads were added to each tube and mixed thoroughly by pipetting. After mixing, tubes were incubated at room temperature for 5 minutes in a rotator mixer. Post incubation, tubes were spun down and kept on a magnetic rack and washed with freshly prepared 70% ethanol. Pellet was resuspended in 20 µl nuclease-free water. After 5 minutes of incubation, RNA was collected from the magnetic rack into a fresh DNA LoBind tube. Further, this RNA was used for RNA adapter ligation.

RNA adaptor ligation and RNA Elution

Eluted RNA was mixed with the 8.0 µl of NEBNext Quick Ligation Reaction Buffer, 6.0 µl of RNA adapter (RMX), 3.0 µl of nuclease-free water, and 3.0 µl T4 DNA Ligase. The reaction mixture was mixed using pipetting thoroughly. The reaction mixture was incubated again at

room temperature for 15 minutes. Post incubation, 40 µl of well-resuspended RNAClean XP beads were added to the reaction and incubated on a rotor mixer for the next 5 minutes. After incubation, the sample was spun down and pelleted down using a magnetic rack. The pellet was washed twice using wash buffer (WSB), and finally, RNA was eluted in 21 µl elution buffer in a DNA LoBind tubes.

Flow cell priming and sample loading

Flow cell priming solution was made using the standard manufacturer's protocol. Precisely, 30µl of thawed and mixed Flush Tether (FLT) was directly added to the tube of thawed and mixed Flush Buffer (FB), and mixed by pipetting up and down. 800 µl of priming mix was added to the priming port and left for 5 minutes. After 5 minutes, 20 µl RNA was mixed with 17.5 µl of nuclease-free water and 37.5 µl of RNA Running Buffer (RRB), and 75 µl sample was loaded on the spot on Nanopore, and run was performed for 48h.

Nanopore Basecalling and Data Analysis

Data was basecalled using the guppy software, and for quality control, we used FastQC to check all the parameters in both control and Infected samples. To check the circRNA in nanopore Direct RNA seq (DRS) we developed a custom script that converts the linear junction reads into the backspliced junction reads. Nanopore library was loaded onto the flow cell (FLO-MIN106) and data was generated. Nanopore raw data was basecalled using Guppy (v 3.2.10), keeping high-accuracy basecalling. The filtered data were mapped to the human genome (hg38) using a custom command-line Blat software package. For capturing the circRNA from the DRS Nanopore reads, we designed a virtual backspliced junction library of all circRNA present in the circBase and circAtlas databases.The circRNA sequences were downloaded from circBase (hg19) and circAtlas(hg38) databases, respectively. Fasta sequences were converted into backsplice fasta sequence using the circDR-seq custom script.Then these backsplice sequence are shortened to 100bp, 50bp upstream, and 50bp downstream to the backsplice junction. This shortening is done mainly to easily find the backsplice read in the Nanopore reads. Further, using pblat, backsplice sequences were aligned to the sequencing reads (generated in this study). pblat is able to map the sequences across the reads, but when it encounters a backsplicing junction, it gets split into two segments that are individually mapped to the different regions of the same gene. Two segments of a read that match upstream and downstream of a splice site signifies a back-splice junction and, therefore, can be taken as a circRNA. To qualify as a circRNA, the Blat score has to reach 60 and should be appropriately aligned 40 bp across the junction (20 upstream and 20 downstream). The circRNAs analysis so far was limited to circbase and circAtlas, which makes it restricted to the annotated circRNAs. We, therefore, expanded our analysis beyond the public database (circBase and circAtlas) which we may have missed. To comprehensively understand the novel circRNA, we prepared a library of all possible exonic combinations of a gene that may lead to backspliced junction. However, this library was limited to genes possessing up to 20 exons. We downloaded all the exonic coordinates from the Ensembl biomart, which then were converted into fasta sequence using bedtools. Next, we joined all possible combinations of exons in a gene and prepared an exonic circRNA library with the help of circDR-seq custom script to identify the backsplice junctions from our DRS Dataset.

Comparison of different pipelines with circDR-seq

Motivated by the fact that we can detect novel backsplice junctions in their native form from DRS Dataset that we generated, we decided to check the robustness of this approach by using a

publically available dataset. For this, we circDR-seq (this paper) compared with a most recently developed tool called “CIRI-long” (13) and circNICK-LRS (15) and checked the overlap between the pipelines.

Supplementary Table: reagents and software source table.

**REAGENT/RESOURCE**	**Source**	**Identifier/Remarks/Purpose**
Antibodies		
Rabbit anti-βactin	Li-COR Biosciences	926-42210
Mouse anti-SRSF1	Thermo Fisher Scientific	324500
Rabbit polyclonal anti-GFP	ABio laboratories	ABio anti-GFP
Mouse anti-BrdU	Sigma-Aldrich	B8434
Mouse anti-HA	Biolegend	901513
Goat anti-mouse IR dye 680	Li-COR Biosciences	925-68070
Goat anti-rabbit IR dye 800	Li-COR Biosciences	925-32211
Mouse anti-HIV-1 P24	NIH, ARP	Reagent#1238
Mouse anti-HIV-1 Tat	NIH, ARP	Reagent#7377
Human anti-CD4-APC	Miltenyi Biotec	130-113-812
Human anti-CD3-FITC	Miltenyi Biotec	130-113-690
Chemicals and Media		
DMEM	Gibco, USA	12100046
RPMI-1640	Gibco	23400-021
Fetal Bovine Serum (FBS), Certified, Performance tested. Origin: United States	Gibco, USA	10082-147
L-Glutamine 200mM	Gibco, USA	25030-081
Pen-Strep (Penicillin Streptomycin)	Gibco, USA	15140-122
Hoechst 33342	Sigma Aldrich	14540
OptiMEM	Gibco, USA	S18531L0102
PBS	HyClone, USA	SH30256.02
Tris(2-carboxyethyl) phosphine hydrochloride (TCEP)	Sigma Aldrich	75259
2xcOmplete^{TM}, EDTA-free Protease inhibitor cocktail	Sigma Aldrich	11873580001
OdysseyBlockingBuffer	Li-Cor	P/N 927-50003

Biotin	Thermo Fisher Scientific	29129
Lipofectamine 3000	Thermo Fisher Scientific	L3000008
Histopaque	Sigma-Aldrich	10771
Agencourt AMPure XP beads	Beckman Coulter	A63881
RNase R	Epicentre	RNR07250
a-Amanitin	Sigma Aldrich	B2263
Raltegravir	NIH, ARP	Reagent#11680
dNTPS	New England Biolabs	N0447
SuperScriptIII RT	Thermo Fisher Scientific	18080044
Quick Ligation Buffer	New England Biolabs	B6058
T4DNA ligase	New England Biolabs	M0202
Critical commercial assays, components, and kits		
RNA Clean and Concentrator-5	Zymo Research	R1016
Qubit HS dsDNA kit	Thermo Fisher Scientific	Q32851
Qubit HS RNA kit	Thermo Fisher Scientific	Q32852
NEBNext rRNA depletion kit	New England Biolabs	E7405
NEBNext poly(A) mRNA Magnetic Isolation module	New England Biolabs	E7490S
NEBNext Magnesium RNA Fragmentation Module	New England Biolabs	E6150S
Nucleospin Gel and PCR clean-up kit	Macherey-Nagel	740609

NucleoSpin Plasmid Transfection-grade, Mini kit for ultrapure plasmid DNA	Machery-Nagel	740490
NucleoBond Xtra Midi EF, Midi kit for endotoxin-free plasmid DNA	Machery-Nagel	740420
NucleoSpin Tissue, Mini kit for DNA from cells and tissue	Machery-Nagel	740952
MyOne streptavidin beads	Thermo Scientific	65001
Flow cell wash kit	Nanopore technologies	EXP-WSH004
FLow cells	Nanopore technologies	FLO-MINSP6
MinION	Nanopore technologies	MinION Mk1B
Direct RNA sequencing kit	Nanopore technologies	SQK-RNA002
Flow cell priming kit	Nanopore technologies	EXP-FLP002
Nuclease free water	Thermo Fisher Scientific	AM9937
Software and algorithms		
Guppy		https://nanoporetech.com
GraphPad Prism	GraphPad Software	https://graphpad.com
R	v3.6.3	https://www.r-project.org
pblat	v2.5	https://github.com/icebert/pblat
Bedtools	v2.27.1	https://bedtools.readthedocs.io/en/latest
samtools	v1.9	http://www.htslib.org
NanoSim	3.0.0	https://github.com/bcgsc/NanoSim
Nanom6A	v2.0	https://github.com/gaoyubang/nanom6A
csvtk	v0.23.0	https://github.com/shenwei356/csvtk
BBMap	v38.81	https://sourceforge.net/projects/bbmap
Biorender	NA	https://biorender.com
Biomart	Ensembl Genes 103	https://www.ensembl.org/biomart
Plasmids		
1. pcDNA 3.1 BS (-)	(64)	A modified pcDNA derivative with

		blasticidin selection marker
2. pCDNA3.1(+) circMini (Empty vector used for circularizing given sequence)	(74)	Addgene #60648; used for overexpression of circular RNAs
3. pCDNA 3.1(+) circ Mini ciTRAN (For ciTRAN expression)	This Paper	Expression of ciTRAN
4. pCDNA 3.1(-) SRSF1-HA	This paper	Expression of SRSF1-HA
5. pCDNA-dRRM1 SRSF1-HA (RRM1 domain is deleted keeping the start codon intact)	This paper	Expression of SRSF1 mutant
6. pCDNA 3.1(-) dRRM2 SRSF1-HA (RRM2 domain is deleted)	This paper	Expression of SRSF1 mutant
7. pCDNA 3.1(-) dRS SRSF1-HA (RS domain is deleted)	This paper	Expression of SRSF1 mutant
8. pCasRx (expresses CasRx protein) (dCasRX was amplified from CARPID BASU-dCasRx #addgene 153209 and site directed mutagenesis was performed to reverse the mutation(R239A/H244A/R858A/H863A) for making CasRX active and subcloned in PLentiCRISPR E backbone	This paper	Expression of CasRx protein
9. pXR004-CasRx pre-gRNA cloning backbone	(75)	Addgene#109054
10. pXR004-gcircZC3HAV1	This paper	gRNA expressing vector
11. pXR004-gcircSMAD2	This paper	gRNA expressing vector
12. pXR004-gcircSLC11A2	This paper	gRNA expressing vector
13. pXR004-gcircPRKDC	This paper	gRNA expressing vector
14. pXR004-gcircPPP3C	This paper	gRNA expressing vector
15. pXR004-gcircB4GALT6	This paper	gRNA expressing vector
16. pXR004-gcircARHGEF7	This paper	gRNA expressing vector
17. pXR004-gcircARHGEF12	This paper	gRNA expressing vector
18. pXR004-gciTRAN	This paper	gRNA expressing vector
19. pXR004-gcircSIN3B	This paper	gRNA expressing vector
20. pXR004-gRFP	This paper	gRNA expressing vector
21. pXR004-gLuciferase	This paper	gRNA expressing vector
22. pEGFP-N1	Clontech	Expression of GFP from CMV promoter
23. pMD2.G	Addgene# 12259	Expression of VSV-G
24. pNL4-3 Nef- Env -	(64, 65)	Defective in Nef and Env
25. NLBN zsGreen (HIV-1 zsGreen) (sequence is provided)	Prof. Massimo Pizzato	Modified NL4-3 which is devoid of Env, Nef and ZsGreen is placed in place of Nef

26. pNL4-3 RT-<br>(Sequence is provided)	Prof. Massimo Pizzato	RT mutant
27. pNL4-3 Env- R- Luc<br>(HIV-1 -Luc)	NIBSC (41), (76)	Envelope, Nef and Vpr mutant and Luc is placed in place of Nef
28. pNL4-3 E-R+ Luc	NIBSC (41),(76)	Envelope and Nef mutant and Luc is placed in place of Nef
29. NCA-zsGreen	(64)	MLV reporter
30. NLBN dEnv vpu fs<br>(sequence is provided)	Prof. Massimo Pizzato	NL4-3 where Envelope is deleted and Vpu has frameshift
31. pNL4-3 Env fs Nef fs dProt<br>(sequence is provided)	Prof. Massimo Pizzato (vector sequence is provided)	Env, Nef has frameshift and Protease is deleted
32. NLBN dEnv dRev<br>(sequence is provided)	Prof. Massimo Pizzato (vector sequence is provided)	NL4-3 where Env and Rev has deletion
33. pNef LAI	(64, 65)	Expresses Nef from LAI
34. pHXB2 env	(64)	Expresses Env of HXB2
35. pTag RFP	NA	Clontech
36. pEGFP-C2 Vpr (NL4-3)	This paper	Expression of GFP tagged Vpr
37. pCDNA 3.1(-) Vpr CH040	This paper	Expression of CH040 Vpr
38. pCDNA 3.1 (-) Vpr REJO	This paper	Expression of REJO Vpr
39. pGL3 NL4-3 LTR	This paper	LTR-Luciferase Reporter
40. pGL3 RHPA LTR	This paper	LTR-Luciferase Reporter
41. pGL3 SUMA LTR	This paper	LTR-Luciferase Reporter
42. pGL3 TRJO LTR	This paper	LTR-Luciferase Reporter
43. pGL3 WITO LTR	This paper	LTR-Luciferase Reporter
44. pSP-CPP	This paper	CMV-driven expression of a secretory Cell-penetrating-peptide (CPP)-tagged protein
45. pSP-CPP-RRM2-HA	This Paper	CMV-driven expression of a secretory Cell-penetrating-peptide (CPP)-tagged RRM2-HA
46. pCDNA 3.1(-) HA-Vpr	This Paper	CMV driven expression of HA-tagged Vpr
47. pCDNA 3.1(-) HA-Vpr Q65R	This Paper	CMV driven expression of HA-tagged Vpr mutant Q65R

48. pCDNA 3.1(-) HA-Vpr W54R	This Paper	CMV driven expression of HA-tagged Vpr mutant W54R
49. pCDNA 3.1(-) HA-Vpr R80A	This Paper	CMV driven expression of HA-tagged Vpr mutant R80A
50. pCDNA 3.1(-) HA-Vpr S79A	This Paper	CMV driven expression of HA-tagged Vpr mutant S79A
51. pLG-Tat	(77)	Luciferase expression from HIV-1 clade C LTR and CMV expresses Tat
52. pCDNA Tat	(77)	Expression of HIV-1 Tat
53. pCMV Renilla luciferase	(78)	Expression of Renilla Luciferase

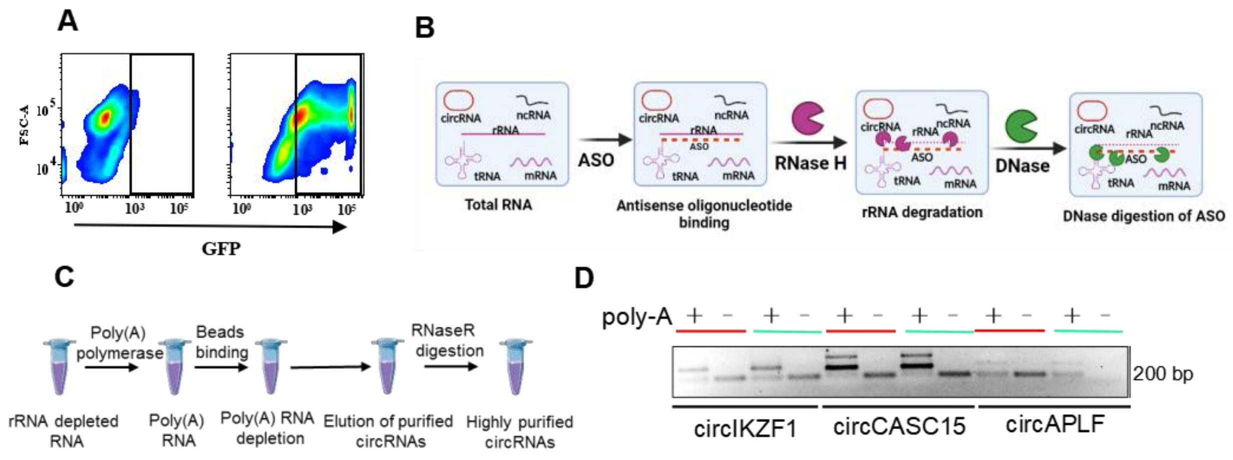

Fig. S1 | Procedures and checks for circRNA enrichment from the infected and mock Jurkat T cells.

(A) Flow-cytometry of mock and HIV-1 zsGreen infected E6.1 T cells. Schematics depicting sequential steps for depleting **(B)** rRNAs by recruiting ASO followed by RNAseH mediated degradation (Refer to supplementary text on circDR-seq for more details), **(C)** linear RNAs by polyadenylation, oligo(dT) magnetic bead-based removal, and RNaseR treatment to enrich circRNA fraction. **(D)** Validation of circRNA fragmentation and addition of polyA by oligo(dT) primed cDNA synthesis and PCR in mock(red) and infected(green) samples.

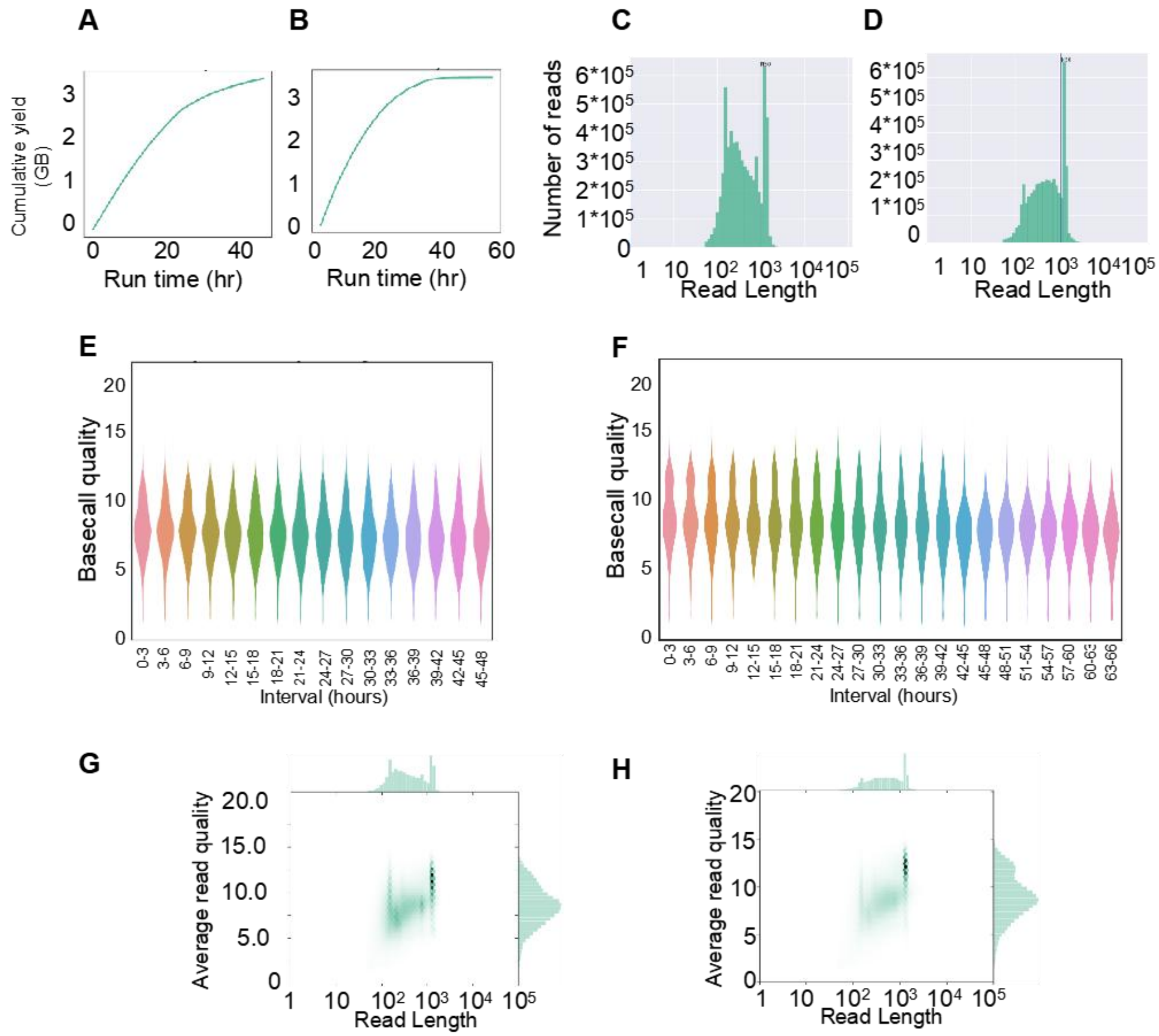

Fig. S2 | Quality checks for Nanopore library and sequencing.
(A, B) the representative cumulative yield is shown as a parameter of flow cell performance over time in mock-treated (A) and infected (B) cells. **(C, D)** Read-length distribution analysis of mock-treated (C) and infected cells (D**). (E, F)** qualitative analysis of base called sequencing reads over time obtained from mock-treated (E) and Infected cells (F**). (G, H)** qualitative analysis of read length and read quality of sequencing reads obtained from mock-treated (G) and Infected cells (H).

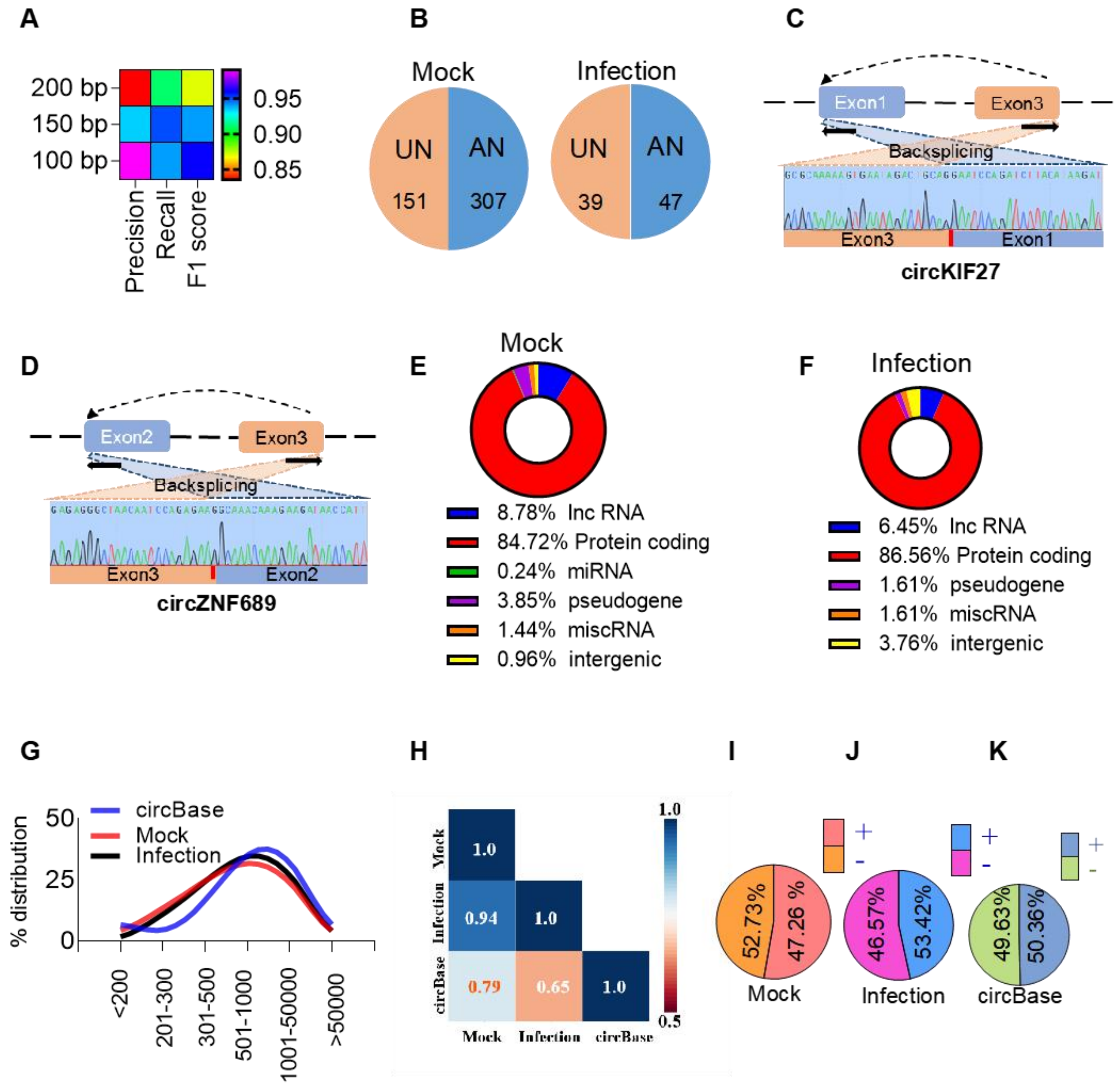

Fig. S3 | Nanopore data analysis, pipeline validation and identification of previously unannotated circRNAs.

(A) Qualitative analysis of various libraries using precision, recall, and F1 score after simulation. **(B)** Unannotated (UN) and annotated (AN) circRNAs in mock and infection. **(C, D)** Validation of unannotated circRNAs (from B) for the presence of novel back-spliced junction by Sanger sequencing. **(E, F)** cataloguing of circRNAs according to the RNA classes **(G)** circRNAs length distribution obtained herein and its comparison with those reported in circBase. **(H)** circRNA length-distribution correlation in mock infection with circBase database. (**I, J, K**) Strand assignment of detected circRNAs (I, J) and its comparison with the circBase database for occurrence (K).

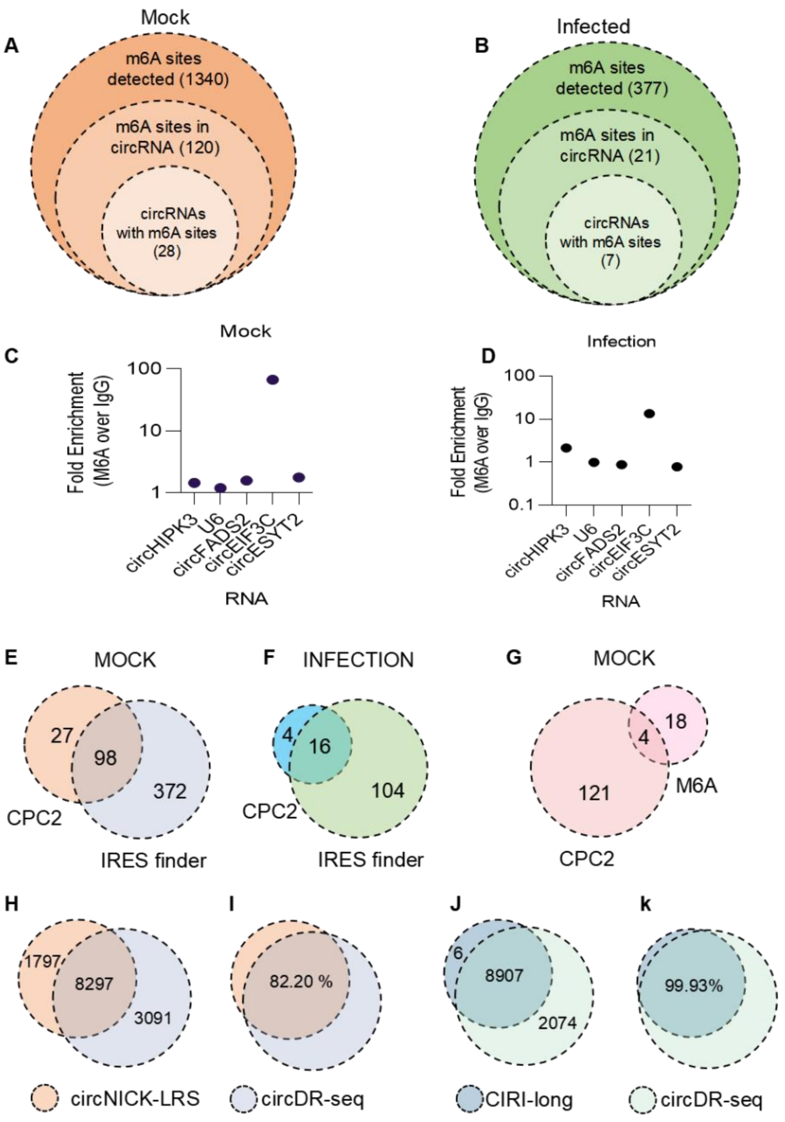

Fig. S4 | Detection of m6A, protein-coding potential of circRNAs, and comparison of circDR-seq with published pipelines.

(**A, B**) Detection of M6A among the reads obtained and M6A presence in the circRNAs. (**C, D**) m6A profile of randomly selected circRNAs from mock and infection revealed by m6A pulldown followed by RT-qPCR. (**E, F**) The protein-coding potential of circRNAs assumed by IRES presence. (**G**) An overlap between M6A modified circRNA with circRNAs having coding potential as estimated using CPC2. (**H, I**) A comparison of circDRseq with circNICK-LRS (Ref#15) and the percentage overlap between circRNA estimation from the data generated for circNICK-LRS (Ref#15). (**J, K**), A comparison of circDRseq with CIRI-long (Ref#13) and the percentage overlap between circRNA estimation from the data generated for the CIRI-long (Ref#13).

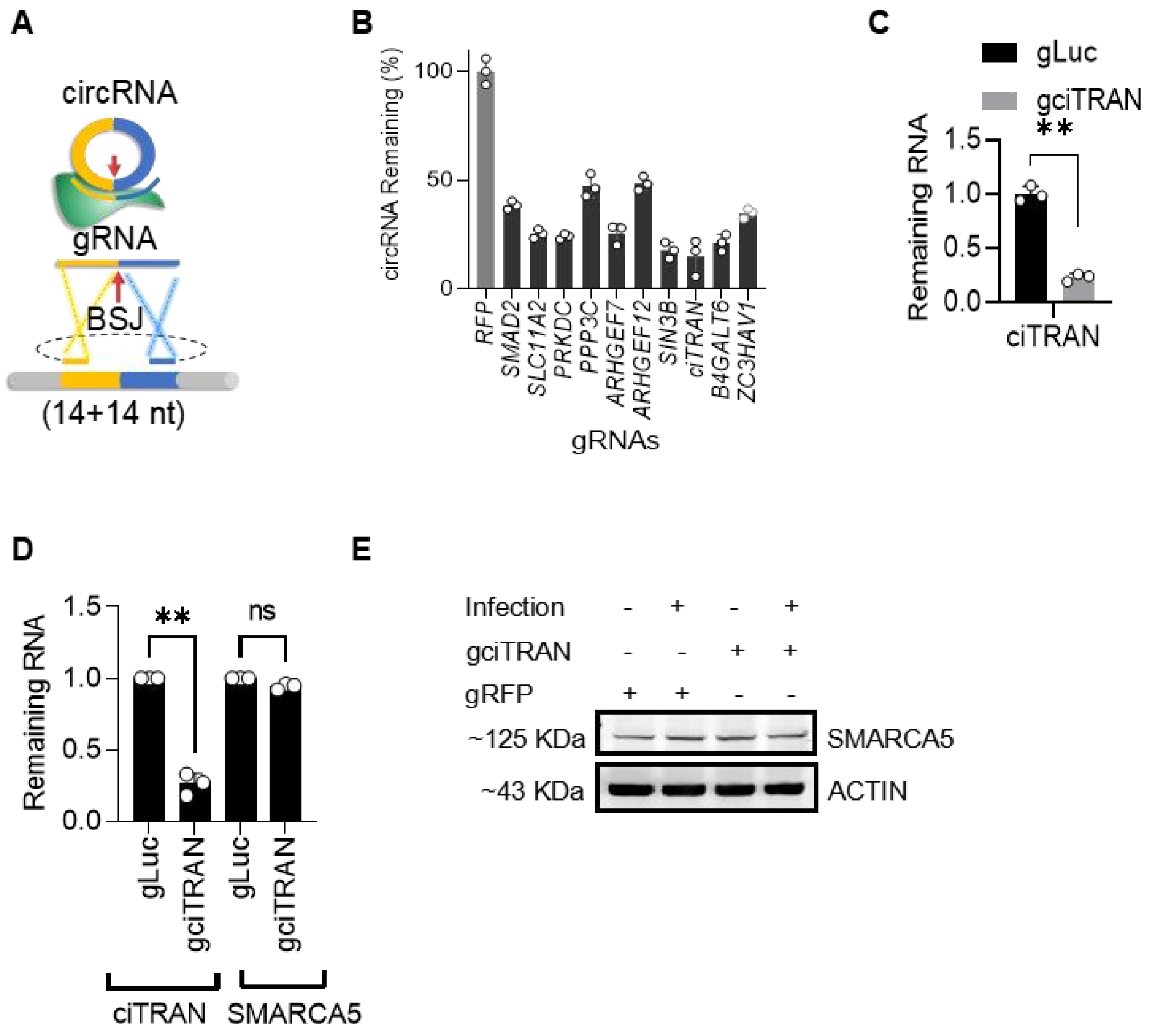

Fig. S5 | CRISPR and ciTRAN gRNA associated specificity checks.
(**A**)gRNA design schematics for a circRNA knockdown by CasRx. **(B)** Levels of circRNAs after CRISPR/CasRx knockdown. **(C)** Levels of ciTRAN after knockdown**. (D)** qRT-PCR analysis of ciTRAN and its cognate linear counterpart (SMARCA5 mRNA) upon ciTRAN knockdown. (**E)** Effect of ciTRAN knockdown on SMARCA5 protein expression in mock and infection.

A

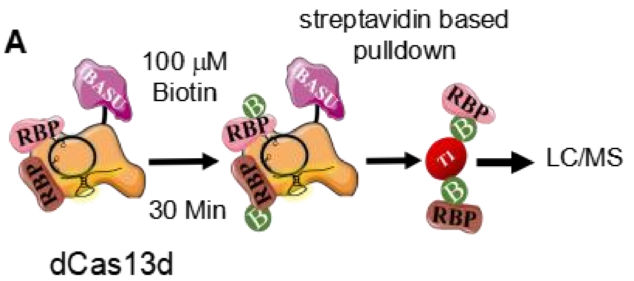

B

Protein identity	Matches	Score
SRSF1	7	44
SIGLEC16	4	27
FAM83D	4	27
NXF2	4	24
KNDC1	4	23
SCAND1	3	23
DNAJC10	4	22
UBE2V1	3	22
ZNF26	4	22
PRAMEF2	4	22

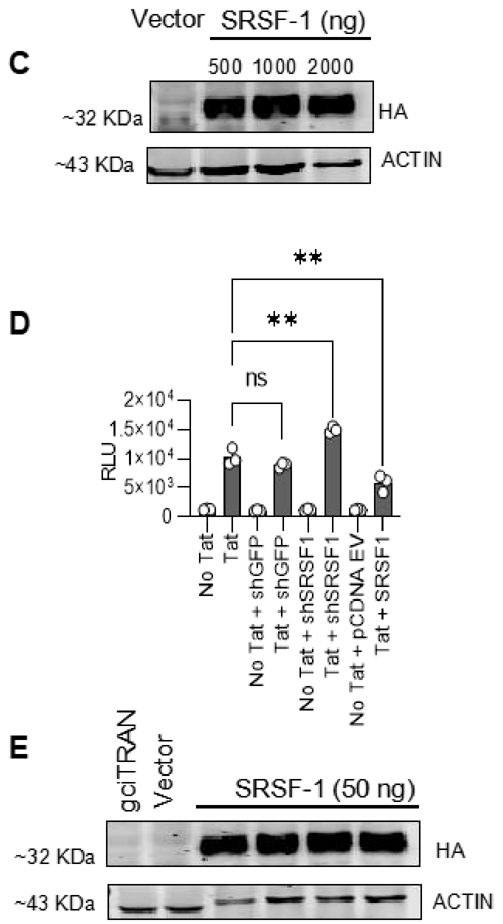

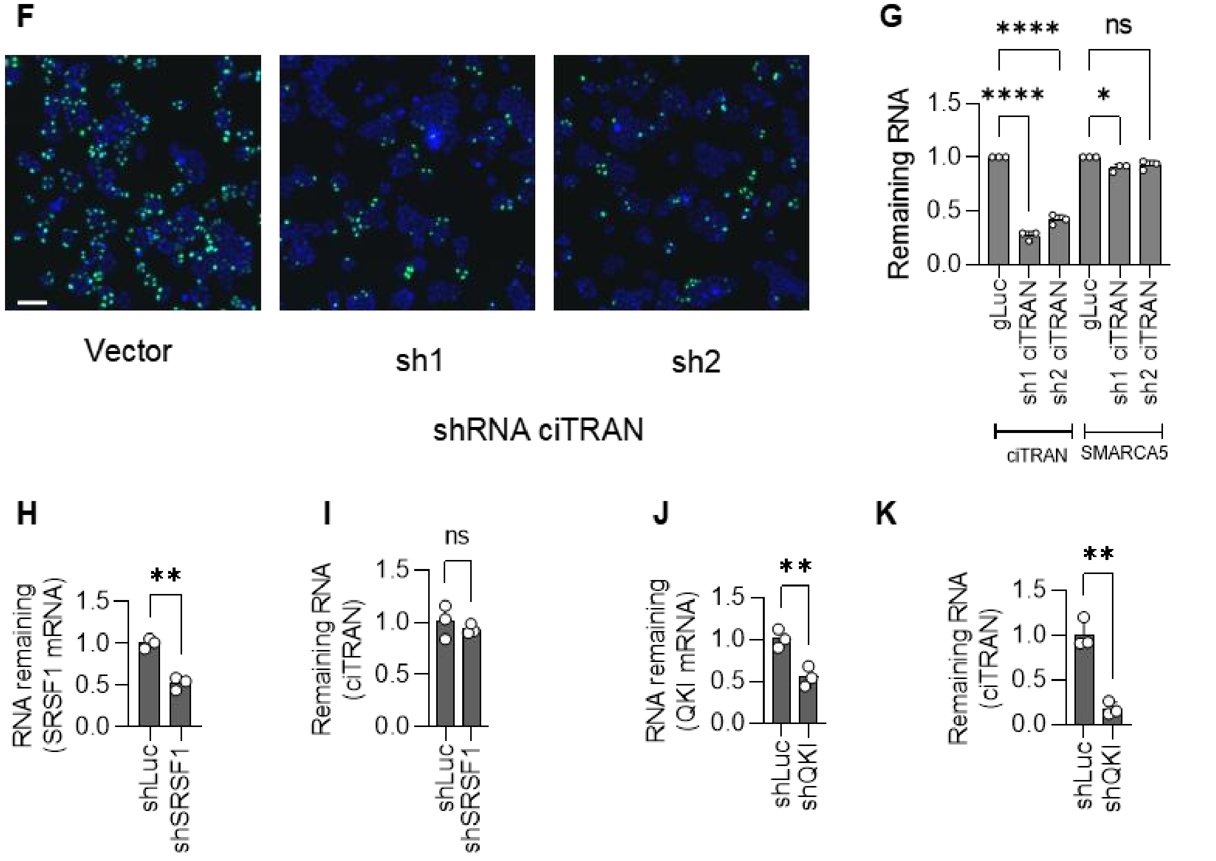

Fig. S6 | CARPID-BASU, LC/MS, SRSF1 levels, shRNA validation and ciTRAN levels in QKI and SRSF1 knockdown cells.

(A) Schematics showing CasRx-BASU-based proximity ligation. **(B)** Proteins identified after LC/MS analysis. **(C)** SRSF1 expression under indicated condition **(D)** Effect of SRSF1 knockdown on Tat-mediated transactivation. LTR-Luc (firefly) construct with and without Tat along with renilla was transfected in either ShLuc or ShSRSF1. Firefly luciferase expression was normalized to renilla luciferase. **(E)** Immunoblotting showing SRSF1 expression under indicated condition. **(F)** Effect of ciTRAN knockdown on HIV-1 infectivity. HIV-1 produced from HEK293T in the presence of either vector, shciTRAN (ciTRAN knockdown cells), were used to infect TZM-GFP reporter cells. Representative images of TZM-GFP target cells (Scale 100μm). **(G)** qRT-PCR analysis of ciTRAN and SMARCA5 RNA upon ciTRAN knockdown by shRNA. (**H-K**) QKI and SRSF1 knockdown validation and their effects on ciTRAN levels.

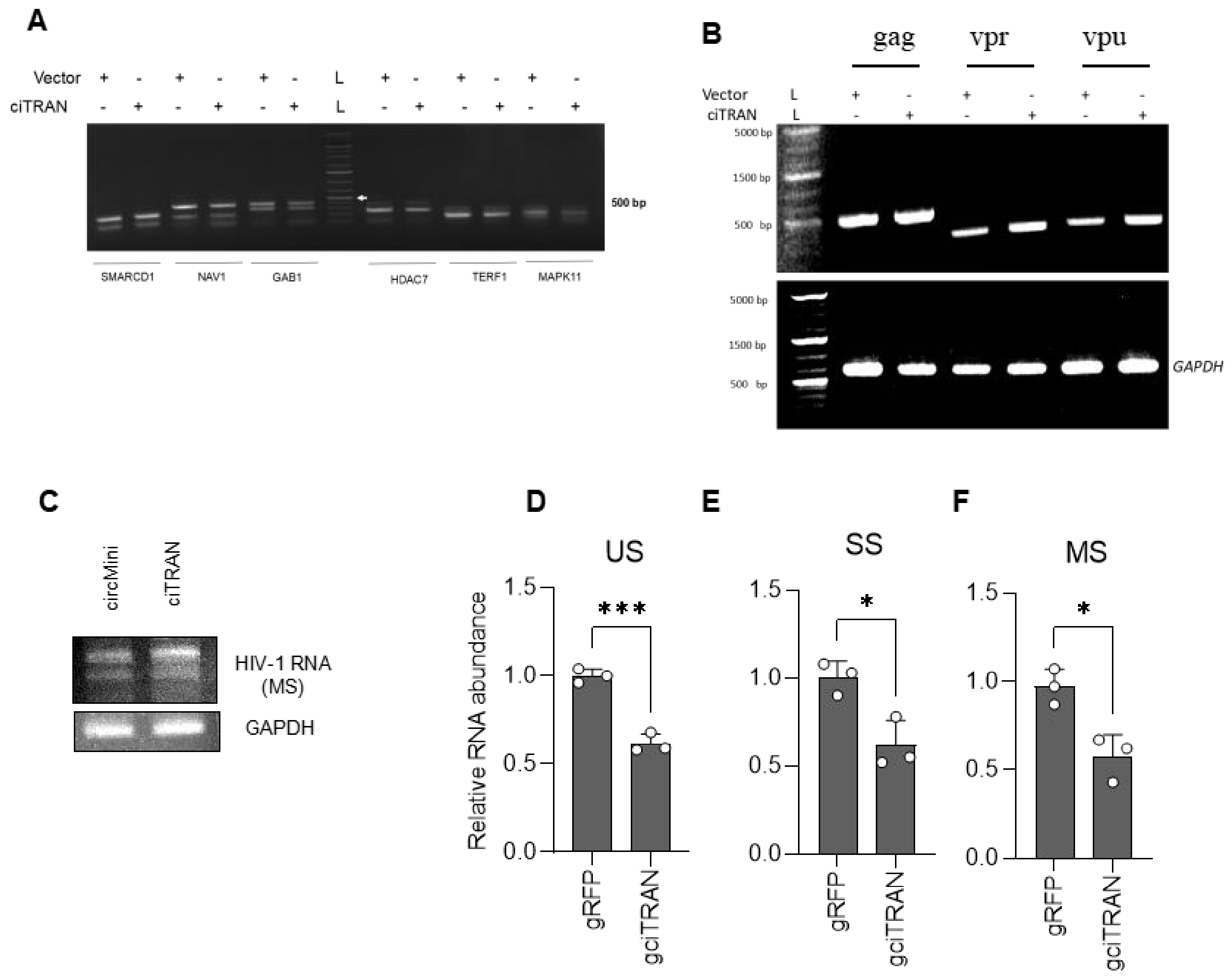

Fig. S7 | Effects of ciTRAN knockdown and overexpression on viral and host RNA splicing. (**A**)Effect of ciTRAN overexpression and concomitant SRSF1 sponging on host RNA splicing. (**B, C**) Effect of ciTRAN overexpression on viral RNA expression (*gag*, *vpr*, *vpu*) and alternative splicing from 2KB multispliced RNA. (**D, E, F**) Effect of ciTRAN knockdown on HIV-1 unspliced, single spliced and multispliced RNA under ciTRAN or RFP knockdown conditions.

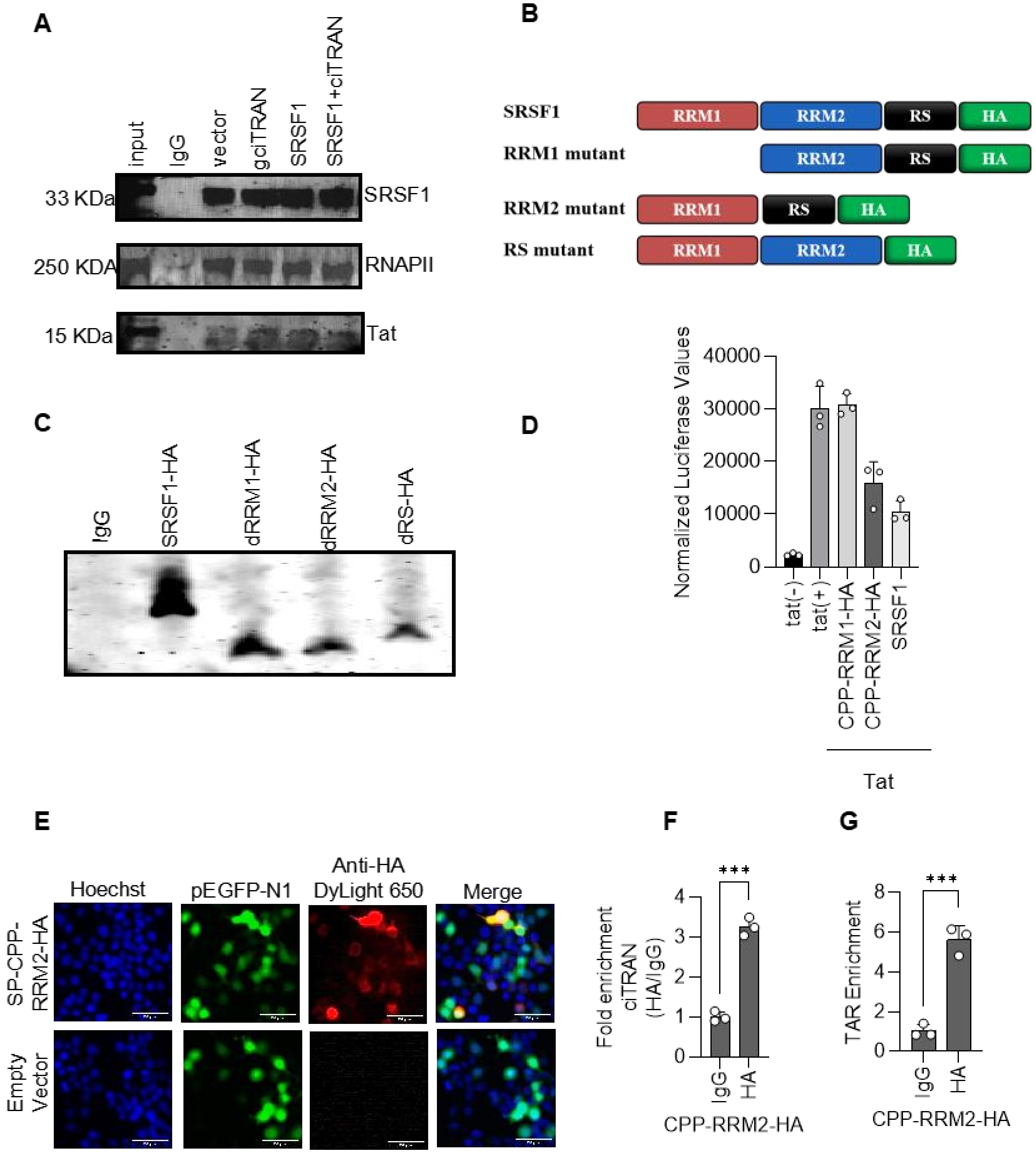

Fig. S8 | SRSF1-, PolII-, Tat -ChIP, SRSF1 deletion mutants, CPP-RRM2 HA ChIP and immunofluorescence and PAR-CLIPs-associated checks.

(**A**)Immunoblotting after ChIP of SRSF1, RNAPII and HIV-1 Tat using specific antibodies. **(B)** Deletion mutants tagged with HA of SRSF1 generated by PCR. **(C)** Immunoblot for SRSF1 mutants using anti-HA antibody after PAR-CLIP enrichment. (**D**) Effect of RRM1-HA and RRM-2 HA on LTR-mediated Luciferase expression. LTR-Luciferase construct was cotransfected with RRM1-HA and RRM2-HA along with Tat and luciferase activity was captured after 48h. (**E**) Immunofluorescence assay to confirm the expression of CPP-RRM2-HA (Scale-bar 50µM). **(F, G)** ciTRAN and TAR association validation with CPP-RRM2-HA by PAR-CLIP and ChIP, respectively.

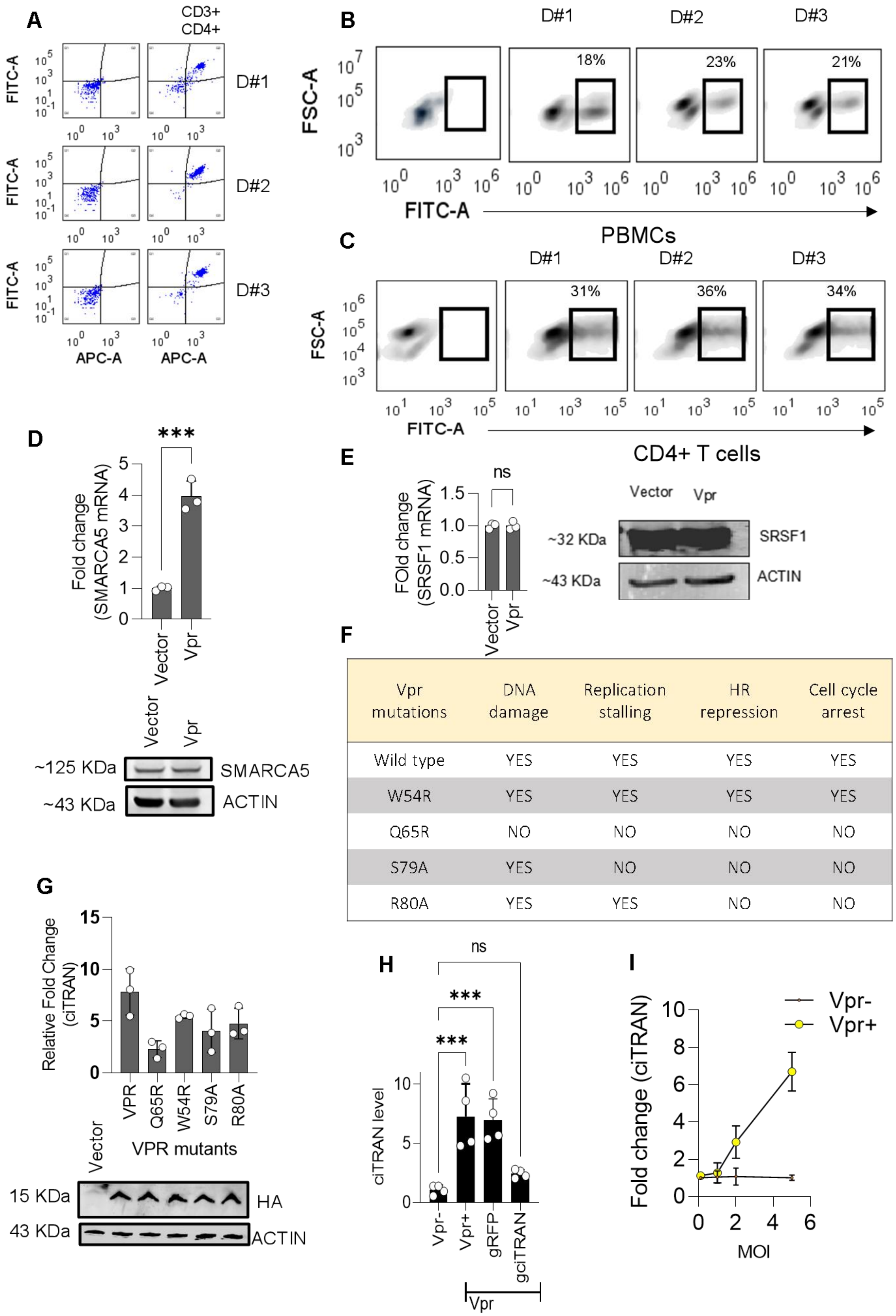

Vpr mutations	DNA damage	Replication stalling	HR repression	Cell cycle arrest
Wild type	YES	YES	YES	YES
W54R	YES	YES	YES	YES
Q65R	NO	NO	NO	NO
S79A	YES	NO	NO	NO
R80A	YES	YES	NO	NO

Fig. S9 | Vpr association, primary cell purification, infectivity and SRSF1 and SMARCA5 level during Vpr expression.

(A) Flow cytometry of $CD3^+/CD4^+$ cells purified from PBMCs of three donors. Infectivity in PBMCs **(B),** and primary $CD4^+$ T cells **(C)** infected using VSVG pseudotyped NLBN zsGreen reporter virus at MOI 5 for 48 h. **(D)** Effect of Vpr expression on SMARCA5 mRNA and SMARCA5 protein. **(E)** Effect of Vpr expression on SRSF1 mRNA and SRSF1 protein. **(F)** details of Vpr and its mutants describing various functions **(G)** Induction of ciTRAN by indicated Vpr mutants and immunoblotting showing expression of respective Wild-type Vpr and its mutant counterparts. **(H)** ciTRAN level induced by Vpr across different conditions and CRISPR-mediated knockdown validation. **(I)** THP-1 infection at different MOIs and ciTRAN induction.

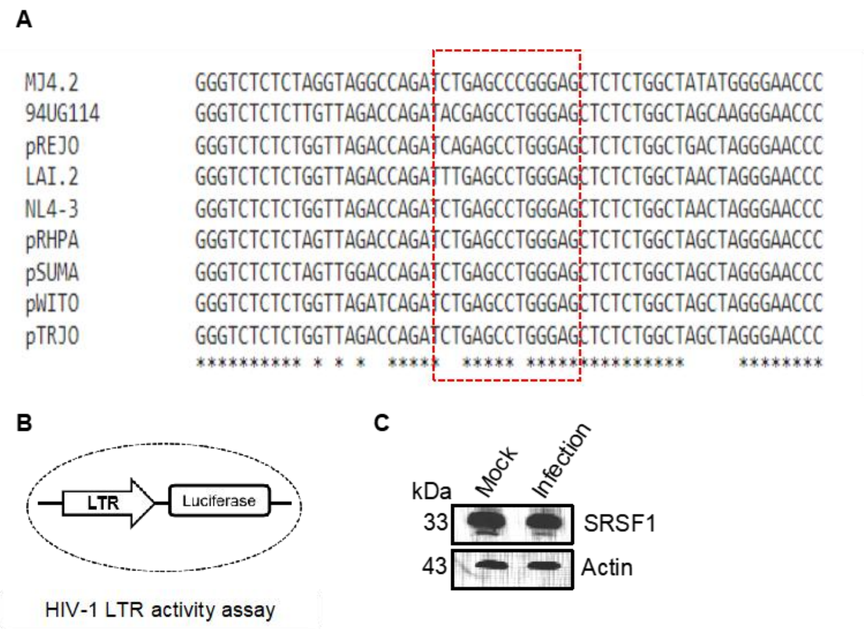

D

circRNA	Mock	Inf	ciTRAN (100 ng)	ciTRAN (150 ng)	ciTRAN (200 ng)
ciTRAN	~37	~139	~164	~262	~417

Fig. S10 | Conservation of SRSF-1 binding site across LTRs of different clades and ciTRAN copy number analysis.

(A) SRSF1 binding site in the LTRs across different clades and transmitted-founder viruses. **(B)** LTR-luciferase reporter minigene construct (PGL3 backbone). **(C)** Effects of infection on SRSF1 expression levels. Actin served as a loading control. **(D)** copy number estimation of ciTRAN using splint-based ligation in mock, infection, and overexpression conditions.

Auxiliary Supplementary Materials for this manuscript include the following:
Legends for auxiliary files 1-9
Auxiliary data table S1: Sequences of oligos and plasmids used in this study.
Auxiliary data table S2: List of circular RNAs detected by circDR-seq using circBase virtual library**.**
Auxiliary data table S3: List of circular RNAs detected by circDR-seq using circAtlas virtual library in mock and infected samples.
Auxiliary data table S4: List of circular RNAs detected by circDR-seq using inhouse exonic virtual library in mock and infected samples.
Auxiliary data table S5: M6A profile of circular RNAs detected using circDR-seq in mock and infected samples.
Auxiliary data table S6: IRES profiling of circular RNAs detected by circDR-seq in mock and infected samples.
Auxiliary data table S7: CPC2 profiling of circular RNAs detected by circDR-seq in mock and infected samples.
Auxiliary File S8: Western blots resource data related to main or supplementary figures.
Auxiliary File S9: Details of patient samples

REFERENCES AND NOTES

1. L. Yan, Y. G. Chen, Circular RNAs in immune response and viral infection. *Trends Biochem. Sci.* **45**, 1022–1034 (2020).

2. C. X. Liu, X. Li, F. Nan, S. Jiang, X. Gao, S. K. Guo, W. Xue, Y. Cui, K. Dong, H. Ding, B. Qu, Z. Zhou, N. Shen, L. Yang, L. L. Chen, Structure and degradation of circular RNAs regulate PKR activation in innate immunity. *Cell* **177**, 865–880.e21 (2019).

3. X. Li, C. X. Liu, W. Xue, Y. Zhang, S. Jiang, Q. F. Yin, J. Wei, R. W. Yao, L. Yang, L. L. Chen, Coordinated circRNA biogenesis and function with NF90/NF110 in viral infection. *Mol. Cell* **67**, 214–227.e7 (2017).

4. P. Xia, S. Wang, B. Ye, Y. du, C. Li, Z. Xiong, Y. Qu, Z. Fan, A Circular RNA protects dormant hematopoietic stem cells from DNA Sensor cGAS-mediated exhaustion. *Immunity* **48**, 688–701.e7 (2018).

5. A. Choudhary, P. Madbhagat, M. Sreepadmanabh, V. Bhardwaj, A. Chande, Circular RNA as an additional player in the conflicts between the host and the virus. *Front. Immunol.* **12**, 602006 (2021).

6. A. Buratin, M. Paganin, E. Gaffo, A. Dal Molin, J. Roels, G. Germano, M.T. Siddi, V. Serafin, M. de Decker, S. Gachet, K. Durinck, F. Speleman, T. Taghon, G. te Kronnie, P. van Vlierberghe, S. Bortoluzzi, Large-scale circular RNA deregulation in T-ALL: Unlocking unique ectopic expression of molecular subtypes. *Blood Adv.* **4**, 5902–5914 (2020).

7. E. Gaffo, E. Boldrin, A. D. Molin, S. Bresolin, A. Bonizzato, L. Trentin, C. Frasson, K.-M. Debatin, L. H. Meyer, G. te Kronnie, S. Bortoluzzi, Circular RNA differential expression in blood cell populations and exploration of circRNA deregulation in pediatric acute lymphoblastic leukemia. *Sci. Rep.* **9**, 1–12 (2019).

8. L. L. Chen, The expanding regulatory mechanisms and cellular functions of circular RNAs. *Nat. Rev. Mol. Cell Biol.* **21**, 475–490 (2020).

9. W. Chia, J. Liu, Y. G. Huang, C. Zhang, A circular RNA derived from DAB1 promotes cell proliferation and osteogenic differentiation of BMSCs via RBPJ/DAB1 axis. *Cell Death Dis.* **11**, 1–11 (2020).

10. N. Chen, G. Zhao, X. Yan, Z. Lv, H. Yin, S. Zhang, W. Song, X. Li, L. Li, Z. du, L. Jia, L. Zhou, W. Li, A. R. Hoffman, J.F. Hu, J. Cui, A novel FLI1 exonic circular RNA promotes metastasis in breast cancer by coordinately regulating TET1 and DNMT1. *Genome Biol.* **19**, 1–14 (2018).

11. J. Zhang, F. Zhao, Characterizing circular RNAs using nanopore sequencing. *Trends Biochem. Sci.* **46**, 785–786 (2021).

12. Z. Liu, C. Tao, S. Li, M. du, Y. Bai, X. Hu, Y. Li, J. Chen, E. Yang, CircFL-seq reveals full-length circular rnas with rolling circular reverse transcription and nanopore sequencing. *eLife* **10**, e69457 (2021).

13. J. Zhang, L. Hou, Z. Zuo, P. Ji, X. Zhang, Y. Xue, F. Zhao, Comprehensive profiling of circular RNAs with nanopore sequencing and CIRI-long. *Nat. Biotechnol.* **39**, 836–845 (2021).

14. A. Rhoads, K. F. Au, PacBio sequencing and its applications. *Genom. Proteom. Bioinform.* **13**, 278–289 (2015).

15. K. Rahimi, M. T. Venø, D. M. Dupont, J. Kjems, Nanopore sequencing of brain-derived full-length circRNAs reveals circRNA-specific exon usage, intron retention and microexons. *Nat. Commun.* **12**, 1–15 (2021).

16. J. Cocquet, A. Chong, G. Zhang, R. A. Veitia, Reverse transcriptase template switching and false alternative transcripts. *Genomics* **88**, 127–131 (2006).

17. G. Luo, J. Taylor, Template switching by reverse transcriptase during DNA synthesis. *J. Virol.* **64**, 4321–4328 (1990).

18. L. Szabo, J. Salzman, Detecting circular RNAs: Bioinformatic and experimental challenges. *Nat. Rev. Genet.* **17**, 679–692 (2016).

19. D. P. Depledge, I. Mohr, A. C. Wilson, Going the distance: Optimizing RNA-seq strategies for transcriptomic analysis of complex viral genomes. *J. Virol.* **93**, e01342-18 (2019).

20. M. S. Xiao, J. E. Wilusz, An improved method for circular RNA purification using RNase R that efficiently removes linear RNAs containing G-quadruplexes or structured 3′ ends. *Nucleic Acids Res.* **47**, 8755–8769 (2019).

21. J. U. Guo, V. Agarwal, H. Guo, D. P. Bartel, Expanded identification and characterization of mammalian circular RNAs. *Genome Biol.* **15**, 409 (2014).

22. J. O. Westholm, P. Miura, S. Olson, S. Shenker, B. Joseph, P. Sanfilippo, S. E. Celniker, B. R. Graveley, E. C. Lai, Genome-wide analysis of Drosophila circular RNAs reveals their structural and sequence properties and age-dependent neural accumulation. *Cell Rep.* **9**, 1966–1980 (2014).

23. A. Rybak-Wolf, C. Stottmeister, P. Glažar, M. Jens, N. Pino, S. Giusti, M. Hanan, M. Behm, O. Bartok, R. Ashwal-Fluss, M. Herzog, L. Schreyer, P. Papavasileiou, A. Ivanov, M. Öhman, D. Refojo, S. Kadener, N. Rajewsky, Circular RNAs in the mammalian brain are highly abundant, conserved, and dynamically expressed. *Mol. Cell* **58**, 870–885 (2015).

24. P. Glažar, P. Papavasileiou, N. Rajewsky, CircBase: A database for circular RNAs. *RNA* **20**, 1666–1670 (2014).

25. Z. Zhang, L. Q. Chen, Y. L. Zhao, C. G. Yang, I. A. Roundtree, Z. Zhang, J. Ren, W. Xie, C. He, G. Z. Luo, Single-base mapping of m6A by an antibody-independent method. *Sci. Adv.* **5**, eaax0250 (2019).

26. Y. Yang, X. Fan, M. Mao, X. Song, P. Wu, Y. Zhang, Y. Jin, Y. Yang, L. L. Chen, Y. Wang, C. C. L. Wong, X. Xiao, Z. Wang, Extensive translation of circular RNAs driven by N6-methyladenosine. *Cell Res.* **27**, 626–641 (2017).

27. P. Ramdas, A. Chande, SERINC5 mediates a postintegration block to HIV-1 gene expression in macrophages. *MBio* **14**, e0016623 (2023).

28. Z. Li, C. Huang, C. Bao, L. Chen, M. Lin, X. Wang, G. Zhong, B. Yu, W. Hu, L. Dai, P. Zhu, Z. Chang, Q. Wu, Y. Zhao, Y. Jia, P. Xu, H. Liu, G. Shan, Exon-intron circular RNAs regulate transcription in the nucleus. *Nat. Struct. Mol. Biol.* **22**, 256–264 (2015).

29. R. Ashwal-Fluss, M. Meyer, N. R. Pamudurti, A. Ivanov, O. Bartok, M. Hanan, N. Evantal, S. Memczak, N. Rajewsky, S. Kadener, CircRNA biogenesis competes with pre-mRNA splicing. *Mol. Cell* **56**, 55–66 (2014).

30. W. Yi, J. Li, X. Zhu, X. Wang, L. Fan, W. Sun, L. Liao, J. Zhang, X. Li, J. Ye, F. Chen, J. Taipale, K. M. Chan, L. Zhang, J. Yan, CRISPR-assisted detection of RNA–Protein interactions in living cells. *Nat. Methods* **17**, 685–688 (2020).

31. S. J. Conn, K. A. Pillman, J. Toubia, V. M. Conn, M. Salmanidis, C. A. Phillips, S. Roslan, A. W. Schreiber, P. A. Gregory, G. J. Goodall, The RNA binding protein quaking regulates formation of circRNAs. *Cell* **160**, 1125–1134 (2015).

32. J.-X. Du, Y.-H. Luo, S.-J. Zhang, B. Wang, C. Chen, G.-Q. Zhu, P. Zhu, C.-Z. Cai, J.-L. Wan, J.-L. Cai, S.-P. Chen, Z. Dai, W. Zhu, Splicing factor SRSF1 promotes breast cancer progression via oncogenic splice switching of PTPMT1. *J. Exp. Clin. Cancer Res.* **40**, 171 (2021).

33. S. Paz, M. L. Lu, H. Takata, L. Trautmann, M. Caputi, SRSF1 RNA recognition motifs are strong inhibitors of HIV-1 replication. *J. Virol.* **89**, 6275–6286 (2015).

34. S. Paz, A. R. Krainer, M. Caputi, HIV-1 transcription is regulated by splicing factor SRSF1. *Nucleic Acids Res.* **42**, 13812–13823 (2014).

35. D. Li, A. Lopez, C. Sandoval, R. N. Doyle, O. I. Fregoso, HIV Vpr modulates the host DNA damage response at two independent steps to damage DNA and repress double-strand DNA break repair. *MBio* **11**, e00940-20 (2020).

36. C. A. Guenzel, C. Hérate, S. Benichou, HIV-1 Vpr-a still “enigmatic multitasker”. *Front. Microbiol.* **5**, (2014).

37. E. L. Rouzic, N. Belaïdouni, E. Estrabaud, M. Morel, J.-C. Rain, C. Transy, F. Margottin-Goguet, HIV1 Vpr arrests the cell cycle by recruiting DCAF1/VprBP, a receptor of the Cul4-DDB1 ubiquitin ligase. *Cell Cycle* **6**, 182–188 (2007).

38. J.-P. Belzile, G. Duisit, N. Rougeau, J. Mercier, A. Finzi, E. A. Cohen, HIV-1 Vpr-mediated G2 arrest involves the DDB1-CUL4AVPRBP E3 ubiquitin ligase. *PLOS Pathog.* **3**, e85 (2007).

39. E. L. Rouzic, M. Morel, D. Ayinde, N. Belaïdouni, J. Letienne, C. Transy, F. Margottin-Goguet, Assembly with the Cul4A-DDB1DCAF1 ubiquitin ligase protects HIV-1 Vpr from proteasomal degradation. *J. Biol. Chem.* **283**, 21686–21692 (2008).

40. C. M. Miller, H. Akiyama, L. M. Agosto, A. Emery, C. R. Ettinger, R. I. Swanstrom, A. J. Henderson, S. Gummuluru, Virion-associated Vpr alleviates a postintegration block to HIV-1 infection of dendritic cells. *J. Virol.* **91**, e00051-17 (2017).

41. R. I. Connor, B. K. Chen, S. Choe, N. R. Landau, Vpr is required for efficient replication of human immunodeficiency virus type-1 in mononuclear phagocytes. *Virology* **206**, 935–944 (1995).

42. M. Mashiba, D. R. Collins, V. H. Terry, K. L. Collins, Vpr overcomes macrophage-specific restriction of HIV-1 Env expression and virion production. *Cell Host Microbe* **16**, 722–735 (2014).

43. S. T. Chang, P. Sova, X. Peng, J. Weiss, G. L. Law, R. E. Palermo, M. G. Katze, Next-generation sequencing reveals HIV-1-mediated suppression of T cell activation and RNA processing and regulation of noncoding RNA expression in a $CD4^+$ T cell line. *MBio* **2**, e00134-11 (2011).

44. D. Barbagallo, A. Caponnetto, D. Brex, F. Mirabella, C. Barbagallo, G. Lauretta, A. Morrone, F. Certo, G. Broggi, R. Caltabiano, G. Barbagallo, V. Spina-Purrello, M. Ragusa, C. di Pietro, T. Hansen, M. Purrello, CircSMARCA5 regulates VEGFA mRNA splicing and angiogenesis in glioblastoma multiforme through the binding of SRSF1. *Cancer* **11**, 194 (2019).

45. D. Barbagallo, A. Caponnetto, M. Cirnigliaro, D. Brex, C. Barbagallo, F. D’Angeli, A. Morrone, R. Caltabiano, G. Barbagallo, M. Ragusa, C. di Pietro, T. Hansen, M. Purrello, CircSMARCA5 inhibits migration of glioblastoma multiforme cells by regulating a molecular axis involving splicing factors SRSF1/SRSF3/PTB. *Int. J. Mol. Sci.* **19**, 480 (2018).

46. D. Barbagallo, A. Caponnetto, C. Barbagallo, R. Battaglia, F. Mirabella, D. Brex, M. Stella, G. Broggi, R. Altieri, F. Certo, R. Caltabiano, G. M. V. Barbagallo, C. D. Anfuso, G. Lupo, M. Ragusa, C. di Pietro, T. B. Hansen, M. Purrello, The GAUGAA motif is responsible for the binding between circSMARCA5 and SRSF1 and related downstream effects on glioblastoma multiforme cell migration and angiogenic potential. *Int. J. Mol. Sci.* **22**, 1678 (2021).

47. J. A. Greig, T. A. Nguyen, M. Lee, A. S. Holehouse, A. E. Posey, R. V. Pappu, G. Jedd, Arginine-enriched mixed-charge domains provide cohesion for nuclear speckle condensation. *Mol. Cell* **77**, 1237–1250.e4 (2020).

48. P. C. Bevilacqua, A. M. Williams, H. L. Chou, S. M. Assmann, RNA multimerization as an organizing force for liquid-liquid phase separation. *RNA* **28**, 16–26 (2022).

49. M. G.-J. Navarro, S. Kashida, R. Chouaib, S. Souquere, G. Pierron, D. Weil, Z. Gueroui, RNA is a critical element for the sizing and the composition of phase-separated RNA–Protein condensates. *Nat. Commun.* **10**, 3230 (2019).

50. Y. Tay, J. Rinn, P. P. Pandolfi, The multilayered complexity of ceRNA crosstalk and competition. *Nature* **505**, 344–352 (2014).

51. V. Tripathi, D. Y. Song, X. Zong, S.P. Shevtsov, S. Hearn, X. D. Fu, M. Dundr, K. V. Prasanth, SRSF1 regulates the assembly of pre-mRNA processing factors in nuclear speckles. *Mol. Biol. Cell* **23**, 3694–3706 (2012).

52. A. C. Francis, M. Marin, P. K. Singh, V. Achuthan, M. J. Prellberg, K. Palermino-Rowland, S. Lan, P. R. Tedbury, S. G. Sarafianos, A. N. Engelman, G. B. Melikyan, HIV-1 replication complexes accumulate in nuclear speckles and integrate into speckle-associated genomic domains. *Nat. Commun.* **11**, 1–17 (2020).

53. S. de Silva, V. Planelles, L. Wu, Differential effects of vpr on single-cycle and spreading HIV-1 infections in $CD4^+$ T-cells and dendritic cells. *PLOS ONE* **7**, e35385 (2012).

54. L. Yurkovetskiy, M. H. Guney, K. Kim, S. L. Goh, S. McCauley, A. Dauphin, W. E. Diehl, J. Luban, Primate immunodeficiency virus proteins Vpx and Vpr counteract transcriptional repression of proviruses by the HUSH complex. *Nat. Microbiol.* **3**, 1354–1361 (2018).

55. W. C. Goh, M. E. Rogel, C. M. Kinsey, S. F. Michael, P. N. Fultz, M. A. Nowak, B. H. Hahn, M. Emerman, HIV-1 Vpr increases viral expression by manipulation of the cell cycle: A mechanism for selection of Vpr in vivo. *Nat. Med.* **4**, 65–71 (1998).

56. S. Gummuluru, M. Emerman, Cell cycle- and Vpr-mediated regulation of human immunodeficiency virus type 1 expression in primary and transformed T-cell lines. *J. Virol.* **73**, 5422–5430 (1999).

57. T. James, M. R. Nonnemacher, B. Wigdahl, F. C. Krebs, Defining the roles for Vpr in HIV-1-associated neuropathogenesis. *J. Neurovirol.* **22**, 403–415 (2016).

58. M. Lai, J. Chen, The role of Vpr in HIV-1 disease progression is independent of its G2 arrest induction function. *Cell Cycle* **5**, 2275–2280 (2006).

59. K. Höhne, R. Businger, A. van Nuffel, S. Bolduan, H. Koppensteiner, A. Baeyens, J. Vermeire, E. Malatinkova, B. Verhasselt, M. Schindler, Virion encapsidated HIV-1 Vpr induces NFAT to prime non-activated T cells for productive infection. *Open Biol.* **6**, 160046 (2016).

60. R. A. Subbramanian, A. Kessous-Elbaz, R. Lodge, J. Forget, X. J. Yao, D. Bergeron, E. A. Cohen, Human immunodeficiency virus type 1 Vpr is a positive regulator of viral transcription and infectivity in primary human macrophages. *J. Exp. Med.* **187**, 1103–1111 (1998).

61. X. Xu, J. Zhang, Y. Tian, Y. Gao, X. Dong, W. Chen, X. Yuan, W. Yin, J. Xu, K. Chen, C. He, L. Wei, CircRNA inhibits DNA damage repair by interacting with host gene. *Mol. Cancer* **19**, 128 (2020).

62. V. R. K. Yedavalli, C. Chappey, N. Ahmad, Maintenance of an intact human immunodeficiency virus type 1 vpr gene following mother-to-infant transmission. *J. Virol.* **72**, 6937–6943 (1998).

63. N. Agarwal, D. Iyer, S. G. Patel, R. V. Sekhar, T. M. Phillips, U. Schubert, T. Oplt, E. D. Buras, S. L. Samson, J. Couturier, D. E. Lewis, M. C. Rodriguez-Barradas, F. Jahoor, T. Kino, J. B. Kopp, A.

Balasubramanyam, HIV-1 Vpr induces adipose dysfunction in vivo through reciprocal effects on PPAR/GR co-regulation. *Sci. Transl. Med.* **5**, 213ra164 (2013).

64. A. Rosa, A. Chande, S. Ziglio, V. de Sanctis, R. Bertorelli, S.L. Goh, S.M. McCauley, A. Nowosielska, S. E. Antonarakis, J. Luban, F.A. Santoni, M. Pizzato, HIV-1 Nef promotes infection by excluding SERINC5 from virion incorporation. *Nature* **526**, 212–217 (2015).

65. M. Pizzato, A. Helander, E. Popova, A. Calistri, A. Zamborlini, G. Palù, H. G. Göttlinger, Dynamin 2 is required for the enhancement of HIV-1 infectivity by Nef. *Proc. Natl. Acad. Sci. U.S.A.* **104**, 6812–6817 (2007).

66. M. Pizzato, O. Erlwein, D. Bonsall, S. Kaye, D. Muir, M. O. McClure, A one-step SYBR Green I-based product-enhanced reverse transcriptase assay for the quantitation of retroviruses in cell culture supernatants. *J. Virol. Methods* **156**, 1–7 (2009).

67. W. Wu, P. Ji, F. Zhao, CircAtlas: An integrated resource of one million highly accurate circular RNAs from 1070 vertebrate transcriptomes. *Genome Biol.* **21**, 101 (2020).

68. M. Wang, L. Kong, pblat: A multithread blat algorithm speeding up aligning sequences to genomes. *BMC Bioinformatics.* **20**, 28 (2019).

69. C. Yang, J. Chu, R. L. Warren, I. Birol, NanoSim: Nanopore sequence read simulator based on statistical characterization. *Gigascience* **6**, 1–6 (2017).

70. P. Ramdas, V. Bhardwaj, A. Singh, N. Vijay, A. Chande, Coelacanth SERINC2 inhibits HIV-1 infectivity and is counteracted by envelope glycoprotein from foamy virus. *J. Virol.* **95**, e0022921 (2021).

71. K. T. Gagnon, L. Li, B. A. Janowski, D. R. Corey, Analysis of nuclear RNA interference in human cells by subcellular fractionation and argonaute loading. *Nat. Protoc.* **9**, 2045–2060 (2014).

72. A. Gardini, Global Run-On sequencing (GRO-seq). *Methods Mol. Biol.* **1468**, 111–120 (2017).

73. M. Hafner, M. Landthaler, L. Burger, M. Khorshid, J. Hausser, P. Berninger, A. Rothballer, M. Ascano Jr, A.C. Jungkamp, M. Munschauer, A. Ulrich, G.S. Wardle, S. Dewell, M. Zavolan, T. Tuschl, Transcriptome-wide Identification of RNA-binding protein and MicroRNA target sites by PAR-CLIP. *Cell* **141**, 129–141 (2010).

74. D. Liang, J. E. Wilusz, Short intronic repeat sequences facilitate circular RNA production. *Genes Dev.* **28**, 2233–2247 (2014).

75. S. Konermann, P. Lotfy, N. J. Brideau, J. Oki, M. N. Shokhirev, P. D. Hsu, Transcriptome engineering with RNA-targeting type VI-D CRISPR effectors. *Cell* **173**, 665–676.e14 (2018).

76. J. He, S. Choe, R. Walker, P. Di Marzio, D. O. Morgan, N. R. Landau, Human immunodeficiency virus type 1 viral protein R (Vpr) arrests cells in the G2 phase of the cell cycle by inhibiting p34cdc2 activity. *J. Virol.* **69**, 6705–6711 (1995).

77. A. G. Chande, M. Baba, R. Mukhopadhyaya, Short communication: A single step assay for rapid evaluation of inhibitors targeting HIV Type 1 Tat-mediated long terminal repeat transactivation. *AIDS Res. Hum. Retroviruses* **28**, 902–906 (2012).

78. T. Mishra, V. Bhardwaj, N. Ahuja, P. Gadgil, P. Ramdas, S. Shukla, A. Chande, Improved loss-of-function CRISPR-Cas9 genome editing in human cells concomitant with inhibition of TGF-β signaling. *Mol. Ther. Nucleic Acids* **28**, 202–218 (2022).
