# Supplementary material for: HIV-1 Vpr induces ciTRAN to prevent transcriptional repression of the provirus
Source: Sci Adv. 2023 Sep 6;9(36):eadh9170. doi: 10.1126/sciadv.adh9170 (PMC10482341; doi:10.1126/sciadv.adh9170)
Supplement: Supplementary file 3 — Auxiliary data files S1 to S2 [file sciadv.adh9170_auxiliary_data_files_s1_and_s2.zip › adh9170_Auxiliary_data_file_S2.docx]

| **Samples** | **Age**  **(Years)** | **Sex** | **ART status** | **Fold change**  **(ciTRAN)** |
| --- | --- | --- | --- | --- |
| Sample-1 | 40 | Female | Yes | 1.198603093 |
| Sample-2 | 36 | Female | Yes | 4.257731571 |
| Sample-3 | 31 | Female | Yes | 13.49361867 |
| Sample-4 | 39 | Female | Yes | 6.653173054 |
| Sample-5 | 57 | Female | Yes | 4.766165748 |
| Sample-6 | 36 | Male | Yes | 4.123256636 |
| Sample-7 | 22 | Male | **No** | 12.12732533 |
| Sample-8 | 42 | Male | Yes | 6.022548967 |
| Sample-9 | 30 | Male | Yes | 1.376511224 |
| Sample-10 | 37 | Male | **No** | 4.612246162 |
| Sample-11 | 33 | Female | Yes | 1.212402129 |
| Sample-12 | 35 | Male | Yes | 4.462242031 |
| Sample-13 | 26 | Male | Yes | 4.067617585 |
| Sample-14 | 32 | Female | Yes | 10.36240236 |
| Sample-15 | 36 | Male | Yes | 6.343022446 |
| Median age (years)=36 | | | | |
