# Supplementary material for: HIV-1 Vpr induces ciTRAN to prevent transcriptional repression of the provirus
Source: Sci Adv. 2023 Sep 6;9(36):eadh9170. doi: 10.1126/sciadv.adh9170 (PMC10482341; doi:10.1126/sciadv.adh9170)
Supplement: Supplementary file 3 — Auxiliary data files S1 to S2 [file sciadv.adh9170_auxiliary_data_files_s1_and_s2.zip › adh9170_Auxiliary_data_file_S1.pptx]

## Slide 1
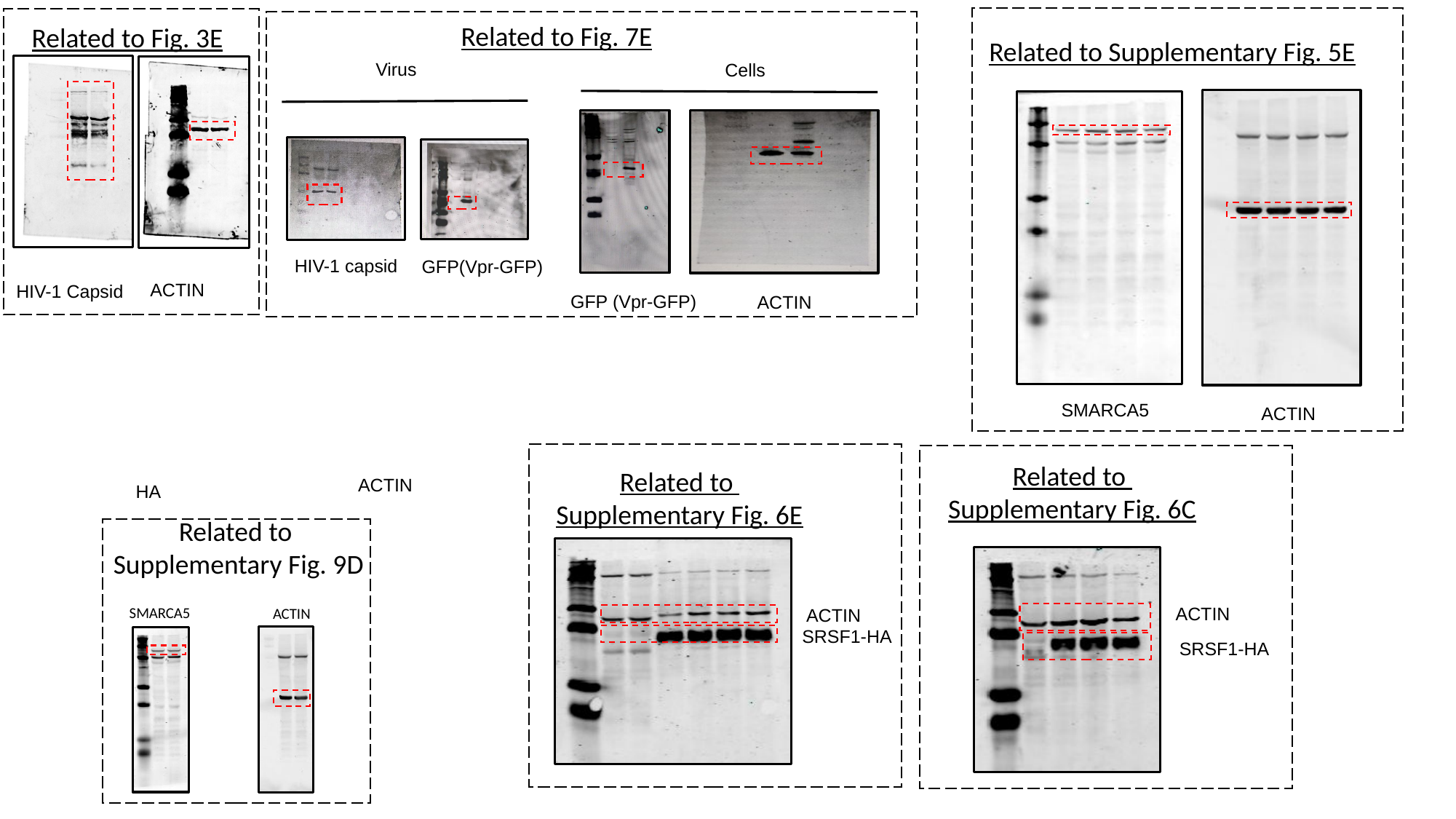

Related to Fig. 7E
Related to Fig. 3E
Related to Supplementary Fig. 5E
Virus
Cells
HIV-1 capsid
GFP(Vpr-GFP)
ACTIN
HIV-1 Capsid
GFP (Vpr-GFP)
ACTIN
SMARCA5
ACTIN
Related to
Supplementary Fig. 6C
Related to
Supplementary Fig. 6E
ACTIN
HA
Related to
Supplementary Fig. 9D
ACTIN
SMARCA5
ACTIN
ACTIN
SRSF1-HA
SRSF1-HA

## Slide 2
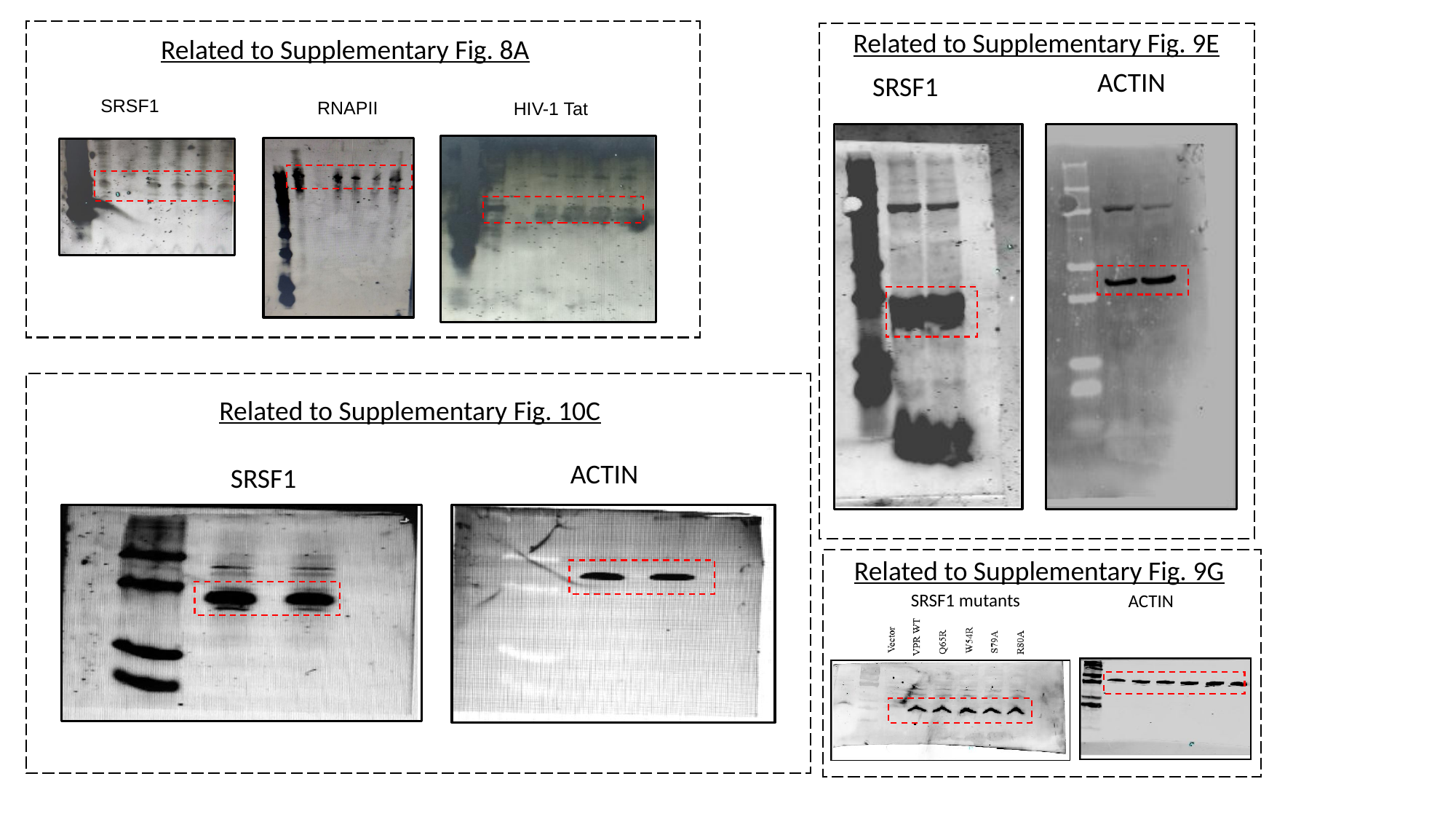

Related to Supplementary Fig. 9E
Related to Supplementary Fig. 8A
ACTIN
SRSF1
SRSF1
RNAPII
HIV-1 Tat
Related to Supplementary Fig. 10C
ACTIN
SRSF1
Related to Supplementary Fig. 9G
SRSF1 mutants
ACTIN
